# SARS-CoV-2 detection and inactivation in water and wastewater: review on analytical methods, limitations and future research recommendations

**DOI:** 10.1080/22221751.2023.2222850

**Published:** 2023-06-21

**Authors:** Parashuram Kallem, Hanaa M Hegab, Habiba Alsafar, Shadi W. Hasan, Fawzi Banat

**Affiliations:** a Center for Membranes and Advanced Water Technology (CMAT), Khalifa University of Science and Technology, Abu Dhabi, United Arab Emirates; b Department of Chemical Engineering, Khalifa University of Science and Technology, Abu Dhabi, United Arab Emirates; c Environmental Health and Safety Program, College of Health Sciences, Abu Dhabi University, Abu Dhabi, United Arab Emirates; d Center for Biotechnology (BTC), Khalifa University of Science and Technology, Abu Dhabi, United Arab Emirates; e Department of Biomedical Engineering, College of Engineering, Khalifa University of Science and Technology, Abu Dhabi, United Arab Emirates; f Emirates Bio-research center, Ministry of Interior, Abu Dhabi, United Arab Emirates

**Keywords:** Wastewater, SARS-CoV-2, detection, inactivation, epidemiology

## Abstract

Severe acute respiratory syndrome coronavirus 2 (SARS-CoV-2) has been detected in wastewater. Wastewater-based epidemiology (WBE) is a practical and cost-effective tool for the assessment and controlling of pandemics and probably for examining SARS-CoV-2 presence. Implementation of WBE during the outbreaks is not without limitations. Temperature, suspended solids, pH, and disinfectants affect the stability of viruses in wastewater. Due to these limitations, instruments and techniques have been utilized to detect SARS-CoV-2. SARS-CoV-2 has been detected in sewage using various concentration methods and computer-aided analyzes. RT-qPCR, ddRT-PCR, multiplex PCR, RT-LAMP, and electrochemical immunosensors have been employed to detect low levels of viral contamination. Inactivation of SARS-CoV-2 is a crucial preventive measure against coronavirus disease 2019 (COVID-19). To better assess the role of wastewater as a transmission route, detection, and quantification methods need to be refined. In this paper, the latest improvements in quantification, detection, and inactivation of SARS-CoV-2 in wastewater are explained. Finally, limitations and future research recommendations are thoroughly described.

## GRAPHICAL ABSTRACT

**Figure:**
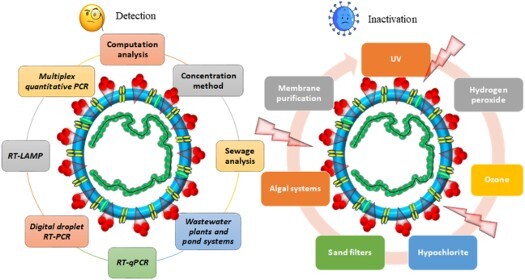

## Introduction

**Figure 1.**
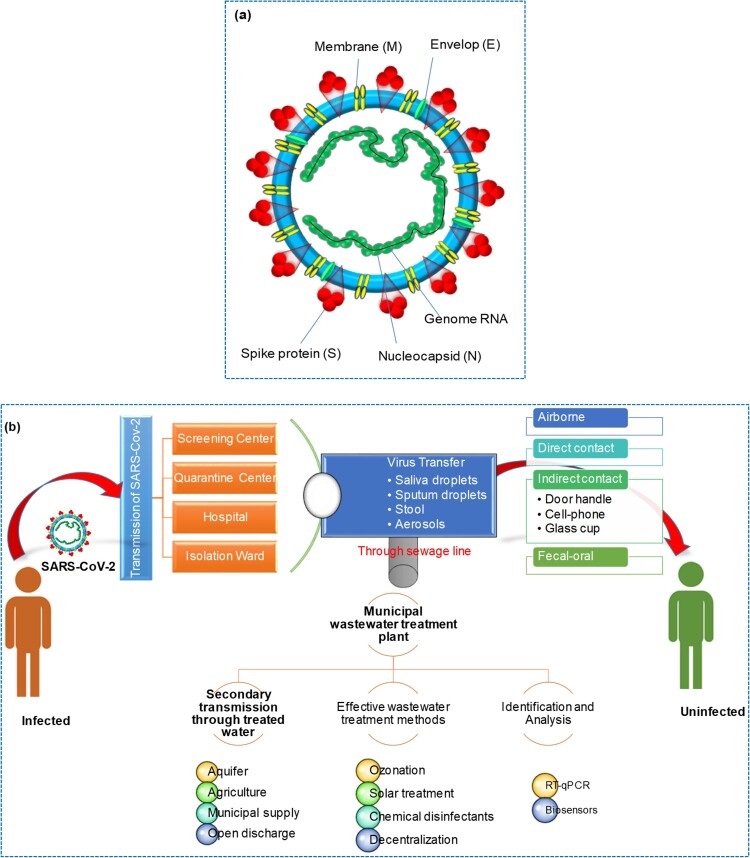
**(a)** SARS-CoV-2 structure. **(b)** SARS-CoV-2 primary and secondary transmission routes.

Severe acute respiratory syndrome coronavirus 2 (SARS-CoV-2) caused a coronavirus disease 2019 (COVID-19) pandemic that has been associated with an international public health crisis. Coronaviruses are a family of RNA viruses that can cause respiratory and other illnesses in humans and animals. There are four main types of coronaviruses: Alpha, Beta, Gamma, and Delta. SARS-CoV and MERS-CoV (Middle East Respiratory Syndrome Coronavirus) are considered to be different lineages of Betacoronaviruses. SARS-CoV emerged in 2002–2003 and caused a global outbreak of severe respiratory illness known as SARS. MERS-CoV was first identified in 2012 and has been responsible for outbreaks of respiratory illness primarily in the Middle East. Alpha coronaviruses primarily infect mammals, including humans and other animals such as bats, while Gamma coronaviruses primarily infect birds [1, 2]. SARS and MERS coronaviruses were highly pathogenic and caused severe respiratory tract infections [3]. China was the first country to report SARS-CoV-2, which is genetically identical to SARS-CoV. SARS-CoV-2 is highly contagious to humans and is usually transmitted by respiratory droplet infection [3]. Coronaviruses are enveloped virions that possess viral envelopes and have a diameter of approximately 120 nm. The crown-like shape on the virus's surface is formed by glycoproteins and proteins, resembling cloverleaf structures, which gives these viruses their name. The nucleocapsid, consisting of capsid-coated proteins, encloses the virus's genetic material. As shown in Figure 1 **(a)**, SARS-CoV-2 comprises four structural proteins, namely nucleocapsid (N), spike (S), membrane (M), and envelope (E). Membrane protein M is crucial in initiating virus entry and envelope formation. Protein E is responsible for virus proliferation, budding, envelope formation, and spread. The multipurpose N protein is responsible for enhancing virus transcription and assembly. Moreover, the Spike (S) protein is responsible for binding the virus to host cells [4].

SARS-CoV-2 can cause both symptomatic and asymptomatic infections in humans. Symptomatic infections are characterized by symptoms including fever, cough, shortness of breath, fatigue, body aches, loss of taste or smell, sore throat, and congestion, among others. Reportedly, infected individuals may have asymptomatic or mild symptoms, making it difficult to control the spread of the virus. Recent global viral outbreaks are not inimitable to SARS-CoV-2. The Spanish flu outbreak was followed by swine flu (H1N1) - the second outbreak of H1N1, which killed 150,000–575,000 people. Globally, as of 11:04 am CEST, 19 April 2023, there have been 763,740,140 reported cases of COVID-19, including 6,908,554 deaths received by WHO from national authorities. Figure S1 (a-b) in supplementary information also shows trends for monthly cumulative COVID-19 cases and deaths, respectively.

Monitoring viral infections in animals can help predict future pandemics and implement viable vaccination programmes. In addition, water and wastewater have been found to contain SARS-CoV-2. To be detected through wastewater surveillance, the pathogen must survive exposure to gastric acid and be excreted in feces. Enteric viruses tend to produce a large number of particles in feces, which makes them easier to detect in wastewater. While SARS-CoV-2 is primarily a respiratory virus, several reports indicate that it is also present in feces, making detection through wastewater testing possible. The viruses are typically excreted into wastewater via feces, urine, saliva, blood, and sputum from COVID-19 patients [5]. A route of transmission for SARS-CoV-2 and its secondary transmission via wastewater is shown in Figure 1 **(b)**.

Wastewater-based epidemiology (WBE) is a viable, efficient, and practical tool for pandemic assessment and management and medical screening for SARS-CoV-2. Monitoring wastewater for signs of pathogens is effective surveillance for many viral infections. Researchers around the world are now pursuing a similar technique for COVID-19 to complement current estimates of disease spread using wastewater data. Monitoring COVID-19 in wastewater is a cost-effective way to track infectious pathogens and transmission dynamics in communities. It provides rapid surveillance of emerging viral infections in human populations and eliminates the need for time-consuming and costly testing of large groups.

## SARS-CoV-2 in water and wastewater: sources and occurrence

During the COVID-19 outbreak, waste management, sanitation, and safe drinking water were critical to protecting human health. Evidence-based and consistently implemented WASH and waste management have prevented the SARS-CoV-2 infection spread. No coronaviruses have been found in surface or groundwater, reducing the risk of infections through drinking water resources. The treated wastewater was found to be free of infectious SARS-CoV-2. Minor amounts of RNA fragments were found in partially treated wastewater [6]. SARS-CoV-2 RNA fragments have been found in untreated sludge and sewage (February and March 2020) due to the increased number of confirmed cases. As the COVID-19 cases decrease, the RNA signal fades substantially. And, this SARS-CoV-2 is enveloped making it low stable in the ecosystem. Other human coronaviruses survived at 20°C in treated water (dechlorinated) and untreated hospital effluent. Coronaviruses were found to have significantly decreased (99.9%) in primary sewage effluents at 23°C, pasteurized settling sewage at 25°C, and reagent grade water at 25°C after two days [7]. These viruses can survive when encased in feces or suspended particulates. When the sewage is flushed, the virus trapped in the water can produce aerosols loaded with the virus, which can spread through the air. Traditional virus concentration methods have also been shown to be ineffective in retrieving encapsulated viruses from water samples collected from the environment. Irrespective of these challenges, one of the first coronavirus identifications in wastewater occurred in 2013 [8]. During the COVID-19 epidemic, samples were collected from Beijing hospital, it is found that SARS-CoV-2 RNA presented 100% and 30% in untreated wastewater and disinfected wastewater samples, respectively [9]. The WBE has demonstrated the effectiveness of lockdown measures to reduce the spread of coronavirus. The presence of SARS-CoV-2 in wastewater was higher than that of confirmed cases, suggesting that multiple genetically distinct clusters were circulating locally. WBE is useful for tracking the spread of human pathogenic viruses and evaluating infection management efforts. However, integration of WBE data into outbreak surveillance programmes will require better knowledge of the characteristics that influence quantification of SARS-CoV-2 RNA in wastewater [1, 10].

Municipal sewage can become contaminated with coronaviruses from various sources, including vomit, sputum, and handwashing. Studies indicate that coronavirus RNA is frequently shed in the feces of infected individuals, which is the primary route of transmission.

**Figure 2.**
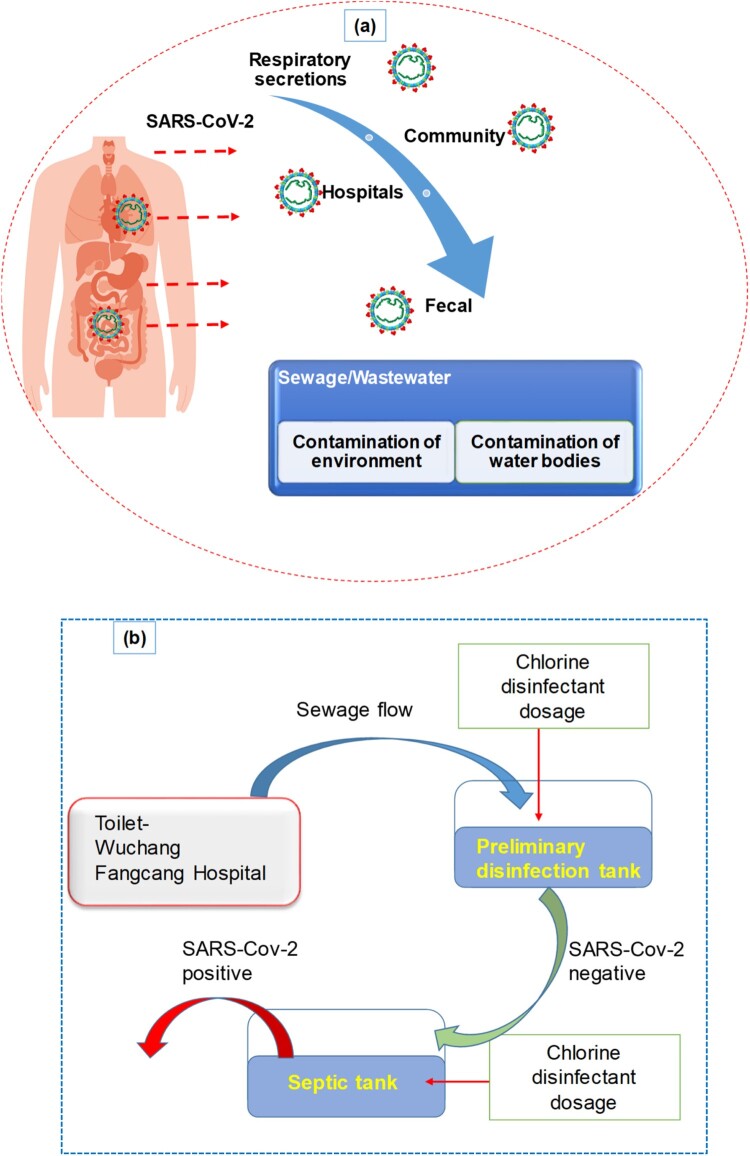
**(a)** Sewage pathways and summary of wastewater infectivity from COVID-19 patients’ secretions and excretions. **(b)** Schematic disinfection procedure of the septic tanks of Wuchang Cabin Hospital.

A summary of the potential infectivity of wastewater and sewage from COVID-19 patients’ secretions and excretions is illustrated in Figure 2 **(a)**. The figure illustrates how SARS-CoV-2 can spread through water from various sources, including hospitals and communities, potentially leading to aerosolization. Hospitals play an important part in the safety of society and medical research activities. The ever-increasing expansion of hospital medication and healthcare operations, as well as the generation and handling of massive amounts of wastewater, presents a huge environmental engineering challenge. Hospital wastewater (HWW) contains a variety of pathogenic microbes that persist even after hospital wastewater was treated. Many pathogenic viruses have been discovered in sewages of HWW treatment plants. It is reported that viruses existed in HWW after dechlorination and UV treatment [11]. The virus settled on the suspended materials and became more stable during wastewater treatment. The SARS coronavirus was detected, and at 20°C it persisted for two days in the HWW of China [9, 12]. A 99.9% reduction in SARS-CoV-2 was detected after 12 days at 23°C. The prevalence of viruses in HWW is a serious source of concern for the environment and public health. Viruses are extremely stable in poor environments, causing a risk to human health. Monitoring and identification of the pollutant are one of the most difficult aspects of HWW management. Municipal wastewater in Spain, sewage samples from Zhejiang University Hospital, and septic tanks from Wuchang Cabin Hospital all tested positive for the presence of SARS-CoV-2 RNA [13, 14].

Figure 2**(b)** demonstrates that septic tanks at Wuchang Cabin Hospital had a notably high concentration of (0.5–18.7) × 103 copies/L of SARS-CoV-2 viral RNA after disinfection with sodium hypochlorite. Septic tanks can release stool particles containing embedded viruses, which can act as a secondary source of SARS-CoV-2 and potentially spread through drainage pipelines. Although overdosing effluents with sodium hypochlorite resulted in negative results for SARS-CoV-2 viral RNA, high levels of disinfection by-product residuals were found, posing significant ecological risks.

[14]. In Australia's wastewater samples, the virus was found in amounts ranging from 1.9–12 copies/100 mL.

According to Tang et al. [4] viral RNA was detected in sputum, nasopharyngeal, and stool samples from an asymptomatic child whose parents tested negative for the virus twice two weeks apart. Another study documented a 6-month-old asymptomatic infant who had close contact with infected parents. The infant tested negative for viral RNA in stool samples on the second day of hospitalization, whereas nasopharyngeal swabs indicated that the infant was both viremic and positive [15]. However, on day nine, a stool sample tested positive, although the infant had no gastrointestinal symptoms. On day seventeen, nasopharyngeal swabs were negative, but another stool sample was not collected.

Kumar et al. [16] reported the first detection of the SARS-CoV-2 gene in wastewater in India. Samples were collected on two separate occasions, May 8 and May 27, 2020, at the Old Pirana Wastewater Treatment Plant (WWTP) in Ahmedabad, Gujarat. This WWTP receives effluent from the Civil Hospital, which treats COVID-19 patients. They used the TaqPath™ Covid-19 RT–PCR kit (Applied Biosystems) to analyze RNAs for the detection of ORF1ab, N-gene, and S-gene of SARS-CoV-2 by RT–PCR. The May 8 and May 27 effluent samples were positive for all ORF1ab, N-protein genes, and S-protein genes analyzed as SARS-CoV-2 genes [16].

SARS-CoV-2 RNA copies were found in varying amounts in wastewater samples from the United States (2 × 10^5^ and 3 × 10^4^ copies/L), France (10^5^–10^6^ copies/L), and Japan (2.4 × 10^3^ copies/L). Detection of SARS-CoV-2 RNA in hospital wastewater samples showed a positive correlation (0.79) with the number of infected patients, highlighting its potential in identifying COVID-19 hotspots. Effective wastewater testing can help identify areas with frequent COVID-19 infections [17]. There is insufficient information about the viruses in treated wastewater and receiving water bodies. In a study by Rimidi et al. [18], it is reported that SARS-CoV-2 RNA was detected in raw wastewater but not in treated wastewater. The isolated virus genome was from the most common strain in Europe. After eight days, the occurrence of RNA in raw wastewater samples was reduced, most likely reflecting the epidemiological trend in the region. The infectivity of the virus was always zero, showing that viral pathogenicity decayed naturally over time after emission. The river samples received (three sites, taken on the same dates as wastewater) exhibited a positive response to real-time RT-qPCR in some cases, most likely due to untreated or inefficiently handled discharges or mixed sewage overflows. However, there was zero in rivers [18].

## Factors influencing the fate of SARS-CoV-2 RNA

The identification of viral RNA is key to WBE for SARS-CoV-2. Several factors may influence the fate of SARS-CoV-2 RNA in wastewater systems. Although biological activity is predicted to be the main cause of the degradation of SARS-CoV-2 RNA in wastewater, chemical deterioration should not be ruled out. Surface waters had a virus degradation rate of 0.07–0.9 per day. The sorption of particulate matter in wastewater and the slime layer on the inside surface of a pipe may impact the mobility of the SARS-CoV-2 RNA in sewer water. The ability of coronaviruses to adsorb on particulate matter in wastewater may affect their survival [7]. The sorption of viruses by solids in solvent or slime environments is determined by virus properties, the sorbent, and the suspension. Solvents have an increased capacity to bind cations and soil particles but dissolved organics reduce this capacity. The influence of sewer biofilms on the decay of coronaviruses in wastewater was studied by Shi et al. [19]. This work revealed that the decay of coronaviruses was faster in the reactors with sewer biofilms than in the reactors without biofilms, indicating a lower risk of transmission of coronaviruses from wastewater to humans.

The fate of viruses in wastewater can be affected by inactivation, degradation, dispersion, and retardation. The most important factor that affects virus survival is temperature. The inactivation rate coefficient increases as the temperature increases, resulting in a shorter viral lifespan in the sewage. Protein denaturation due to increased extracellular enzyme activity resulted in decreased viral survival as the temperature increased [20]. In contrast to enveloped viruses, non-enveloped viruses lasted longer. With an inactivation rate coefficient of about one-tenth of other enveloped viruses, the enveloped SARS-CoV was much more stable and somewhat similar to the non-enveloped virus in the wastewater. The occurrence of other bacteria in wastewater impacts virus survival. Without other microorganisms in wastewater, the virus inactivation rate coefficients vary with temperature. The presence of viruses with temperature was matching the wastewater that was unpasteurized, though the magnitudes of the inactivation rate coefficients were smaller. This suggests that the presence of other bacteria in wastewater has accelerated viral inactivation. The pH affects the viability of viruses in wastewater. The human coronavirus 229E is most stable at pH 6 at 33°C and remains stable through the pH range of 5–8. It remains unclear how the stability of SARS-CoV-2 varies over time, although recent tests have found that it survives in a wide pH range [3–10] without an apparent loss for one hour. The pH of the solution influences the sorption of the virus and its isoelectric point. In solutions with a pH lower than the virus IEP, positive charge-containing virus particles adsorb on the negatively charged surfaces. The hydrophobicity of the virus, a key factor in virus sorption during soil movement, may also affect sorption. The virus-adsorbent interaction can be electrostatic or hydrophobic, depending on pH. The presence and quantity of disinfection chemicals such as residual chlorine could also affect the virus stability. Moreover, the virus was found to be more effectively inactivated by free chlorine than by chlorine dioxide. SARS-CoV is completely inactivated when free residue chlorine exceeds in wastewater [12].

After the first report of SARS-CoV-2 in wastewater, many studies have been published worldwide [21, 22], demonstrating a shift toward estimating the transmission of SARS-CoV-2 through wastewater surveillance. To supplement human COVID-19 surveillance, Sharkey et al. [23] developed a wastewater monitoring programme. The levels of individual building SARS-CoV-2 RNA varied more than those of groups of buildings. The densities on the building and group scales ranged between 10^1^ and 10^6^ gc/L, allowing a logarithmic transformation to reveal trends. The physical–chemical characteristics of wastewater were affected by the water source but not the SARS-CoV-2 levels. Electronegative filters and ultracentrifuge techniques produced similar concentration results. Electronegative filtering was simpler due to the ease of obtaining supplies, which facilitated sample sharing among labs. The use of V2GqPCR simplified the detection and quantification of SARS-CoV-2 in wastewater. The method was developed to quantify the SARS-CoV-2 RNA isolated from wastewater. It was intended to be a fascinating comparison to more mainstream RT-qPCR. Using V2G-qPCR to amplify wastewater-isolated RNA eliminates the need for cDNA synthesis, saving time and money. This new method could get results from sampling to detection in 12 h, which is faster than other methods and could be useful for COVID-19 early detection. Compared to human monitoring data, SARS-CoV-2 wastewater estimates have provided initial indications of COVID-19 outbreaks. An increase in viruses per day in wastewater samples indicated undiagnosed COVID-19 infections. They were found four days after the growth in RNA levels in wastewater was noticed [23]. The ideal lag period would be achieved by sampling wastewater daily rather than weekly to match COVID-19-positive rates. By detecting positive people earlier, researchers would be able to slow the spread of disease.

## Different tools and analytical strategies for COVID-19 detection and their efficiency

While inhalation remains the primary route of viral transmission, making nasopharyngeal swabs the preferred method for identifying viral RNA to monitor the spread of COVID-19, analysis of the virus in wastewater can also provide valuable epidemiologic information. Based on these premises, D’Agostino et al. [24] developed a rapid and accurate SARS-CoV-2 genotyping assay panel that can distinguish between the wild type (Wuhan strain) and the major SARS-CoV-2 strains. The assay can quickly identify the virus in clinical samples, such as RNA obtained from nasopharyngeal swabs, as well as wastewater samples. The proposed approach is a versatile solution that allows the screening of a variable range of samples and can be easily adjusted and adopted to tackle the emergence of new viral strains. By incorporating a pre-amplification step, all customized probes proved to be highly sensitive, even when analyzing complex samples such as wastewater, and enabled detection at a very low limit of detection. The potential of WBE as a potent tool to detect the scope of a disease outbreak or anticipate an emerging one is significant. However, the costs associated with performing PCR-based tests remain a primary obstacle to the more widespread and consistent use of WBE. Consequently, it is crucial to explore and further investigate alternative methods until comprehensive applications for wastewater-based surveillance of SARS-CoV-2 become feasible in developed and low-income countries. Additional research is needed to fully implement these methods for WBE in real-world scenarios [1].

### Computation analysis

**Figure 3.**
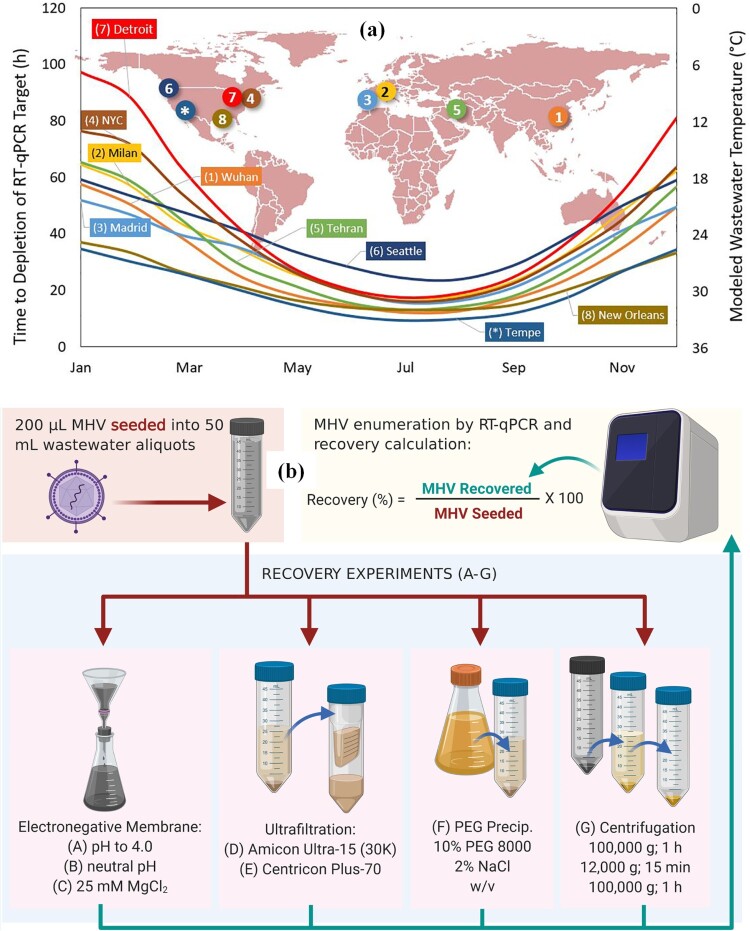
**(a)** Annual change in the time from the sewer to depletion of SARS-CoV-2 calculated for 8 cities (city of Tempe, Arizona, USA) affected by the COVID-19 pandemic (the figure was adapted from Hart et al. [25] with permission). **(b)** Virus concentration methods (the figure was adapted from Ahmed et al. [28] with permission).

The effective management of COVID-19 can be facilitated by computational models developed by a range of professionals, including government officials, researchers, programmers, software engineers, and developers. Various computational- modelling approaches such as fuzzy logic, blockchain, and system dynamics were used during the pandemic to help officials combat the spread of the virus. Through a combination of rigorous testing, contact tracing, and lockdown measures, it is possible to identify asymptomatic individuals and prevent the outbreak of COVID-19 in the population. In addition, computational models can be used to optimize various parameters to control the spread of infection with limited resources. Hart et al, [25] used computational analysis and modelling to investigate the practicality, cost, potential, and limitations of employing WBE to enumerate active coronavirus infections locally and worldwide. Detection of one infected case per 2,000,000 non-infected people in community wastewater is theoretically possible, with some successes currently being recorded from throughout the world. Temperature, sewer travel time, and individual water use were found to be critical factors in computer simulations of historical, current, and emerging epidemic hotspots. Figure 3 **(a)** shows an overview of the sites around the world measured in this study and the estimated temperature of the wastewater throughout the year. WBE was identified as a quick, low-cost, and potentially powerful technique to monitor SARS-CoV-2 as a result of computational modelling and cost analysis. However, the uncertainty of WBE and the impact of the steps associated with the estimation of SARS-CoV-2 prevalence are largely unknown. Li et al. [22] systematically reviewed and estimated the uncertainties associated with estimating SARS-CoV-2 prevalence. Li et al. [26], used data-driven models for the first time to assess the COVID-19 cases using a multi-national WBE data obtained as a result of a literature survey. Three different data-driven models were used in this study, i.e. adaptive neuro-fuzzy inference system (ANFIS), artificial neural network (ANN), and multiple linear regression (MLR). It was found that the ANN and ANFIS models showed better performance than the MLR model.

The analysis and interpretation of sequencing data obtained from wastewater samples continue to present several challenges. One of these challenges is the difficulty of accurately relating estimates obtained from wastewater samples to a specific local population due to human mobility. Collection and sequencing of SARS-CoV-2 material from wastewater pose several challenges to data analysis, including lower sample quality and potential contamination from multiple patients. Analytical workflows must also consider samples from multiple locations and time points and integrate the information in a user-friendly manner. Analysis of the genomic makeup of SARS-CoV-2 in wastewater samples can be difficult because of the low concentration of viral material, PCR inhibitors, fragmented viral genomes, and the presence of other DNA and RNA genomes from human, bacterial, and viral sources. Although it is possible to detect COVID-19 variants early using wastewater samples, the interpretation of individual samples can be problematic. This is because only a small number of signature mutations are typically present at low frequencies initially, making it difficult to distinguish between true signal and noise in the sequencing data. Noise in the data is high due to the technical challenges encountered in sample collection, processing, RNA extraction, and amplification [27].

### Concentration method

For enriching SARS-CoV-2 in wastewater, multiple concentration approaches are now applied (**Table SI in supplementary information**). Although these methods have successfully recovered enteric viruses without envelopes from wastewater, recovery rates for SARS-CoV-2 have not yet been determined. The absence of appropriate controls similar to SARS-CoV-2 was a significant shortcoming of the current methodology, as mentioned. In classical WBE, for example, deuterated and/or 13C surrogates are used to quantify target molecules. Ahmed et al. [28] examined seven different concentration methods were examined, and the recovery of SARS-CoV-2 was calculated in wastewater (Figure 3 **(b)**). Absorption-extraction techniques recover SARS-CoV-2 from wastewater quickly and easily. MgCl_2_ in wastewater resulted in the best electronegative membrane recovery. The Amicon Ultra-15 filter performed admirably. However, the volume of the type of sample analyzed and the filter type impacted the recoveries. Finally, PEG produced low yields, resulting in PCR inhibitor co-concentration. Pre-centrifugation, which is often used in concentration to remove larger particles and trash, may impact recovery, as pellet production may absorb some genetic material. It is worth emphasizing that data on encapsulated viruses are few. More research is needed on existing methods and their recovery efficiency in different types of water.

### Sewage analysis

Sewage contains human health and habits data that can serve as a community health perspective and be used to fine-tune community health reactions to a pandemic. During the re-emergence of polio in 2013, Israel's sewage surveillance system tracked polio in sewage trunk lines, resulting in a rapid response to the epidemic [29]. Although viral shedding differs from person to person and throughout a transmission, the sewage system can combine these differences into a standard that signifies the larger society. The link between COVID-19 propagation and temperature may cause seasonal fluctuations in the viral genetic material in sewage, with exposure points, increased in climatologically favourable zones (4-12°C). As SARS-CoV-2 is a new virus in the sewage system, WBE research is still in its early stages in terms of the development and method validation for virus detection.

### Wastewater treatment plants (WWTP) and pond systems

The WHO emphasized safe wastewater management as some animals may be competent hosts and recycled water may be used for agriculture, limiting the environmental spread of SARS-CoV-2 is critical. Gerba et al. [30] state that wastewater plants must provide a virus reduction factor based on reclaimed water usage. To make the water potable, they claim that a 12-log_10_ removal is required. Regarding recycled water safety, Ahmed et al. [31] used the same elimination targets. Because SARS-CoV-2 is a fatal human virus, the Centres for Disease Control and Prevention (CDC) has issued the Biosafety level 2 (BSL-2) and COVID-19 guidelines that procedures such as precipitation and membrane filtering should be done with unidirectional airflow and BSL-3 safeguards in a BSL-2 lab. Infections are frequently replaced by viral surrogates. The SARS-CoV-2 surrogate virus MHV has been used to study virus concentration approaches for wastewater [28]. Many other animal coronaviruses have been used to study human coronaviruses in wastewater. Some laboratories use MS2 enveloped Escherichia virus or enveloped bacteriophage φ6 for safety reasons. Along with AiV-1 and TMV, PMMoV has been used to assess the reduction of virals in wastewater. Water treatment ponds are widely used around the world because they are the most technically simple solution. 71 wastewater treatment pond systems showed a log_10_ reduction in virus concentration in 14.5 and 20.9 days. The recommended pathogen reduction is 6–7 log_10_ units. This goal can only be achieved by combining multiple strategies. The primary treatment is ponds, with three treatment steps. The pond's chemical constitution and optical characteristics affect the way of virus removal in its treatment system. Virus particles interact with other particles, such as microorganisms, and sunlight-mediated mechanisms appear to be involved in the pond treatment system.

A very sensitive method is quantitative RNA detection to monitor the SARS-CoV-2 virus in wastewater. Viral genetic material is protected from environmental factors by virus particles found in wastewater. The RNA within the compact virus particles does not degrade and can be detected in a laboratory setting. As a result of the infectious nature of the virus, inactivating it should be the first step in all viral RNA methods. Some studies report using personal protective equipment (PPE) during laboratory procedures, disinfecting sample bottles with UV light before handling them in the laboratory, and pasteurizing samples at 60°C for 30–90 min to degrade viral capsids before the experiment [32]. SARS-CoV-2 is not an intestinal virus, it can be detected differently depending on the wastewater sample used. The presence of SARS-CoV-2 in different types of sludge in a Spanish WWTP was investigated by Balboa et al. [33]. It was found that SARS-CoV-2 in wastewater has a strong affinity to biosolids. It was also found that primary sludge (thickened one) is a suitable spot for SARS-CoV-2 due to its long residence time and higher solids concentration.

### RT-qPCR and droplet digital PCR (ddPCR)

Currently, RT-qPCR or (nested) RT–PCR are the most widely used methods for detecting SARS-CoV-2 infections. According to Corman et al. [34], three TaqMan-based quantitative PCR assays were developed to target the genes of the RNA-dependent RNA polymerase (RdRp), the envelope (E), and the nucleocapsid (N) protein. As far as RT-qPCR assays are concerned, the gene encoding the N protein is the most commonly used gene to target.

**Figure 4.**
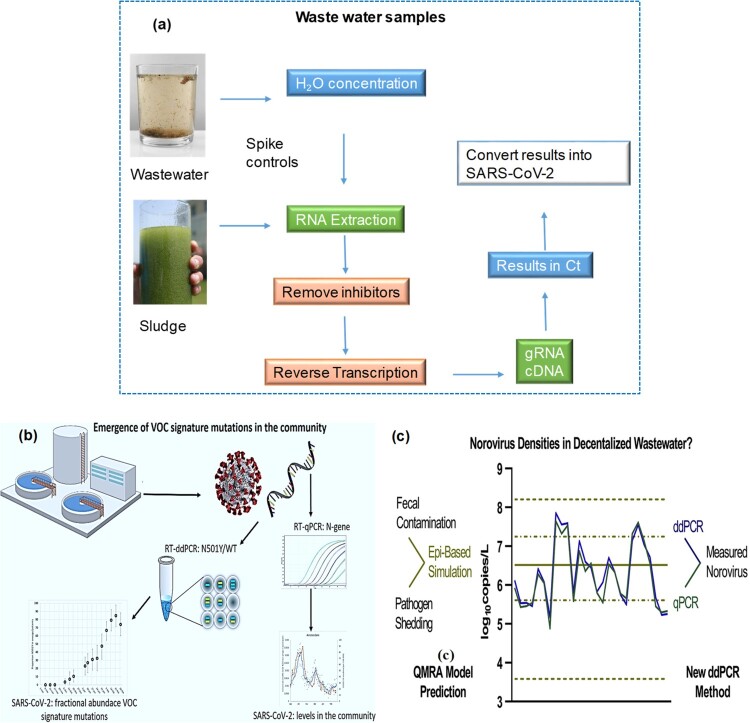
**(a)** General strategy used to collect wastewater or sludge samples and detection of viral RNA using RT-PCR. **(b)** Digital droplet RT-PCR to detect signature SARS-CoV-2 mutations of variants (the figure was adapted from Heijnen et al. [36] with permission). **(c)** Concentrations of Norovirus genogroup II in combined decentralized wastewater collections were determined by qPCR and ddPCR (the figure was adapted from Jahne et al. [37] with permission).

The process involves using specific DNA probes and primers to amplify viral RNA, but RNA cannot be amplified directly and must be first transformed into DNA. This is achieved through the use of “reverse transcriptase (RT)”, giving the technique its name RT qPCR. In summary, the RNA extracted from the wastewater sample is transformed into cDNA (complementary DNA) using reverse transcriptase (RT) and a poly dT primer, which binds to RNA containing the polyadenylated tail and converts it to DNA, as shown in Figure 4 **(a)**. The DNA is then amplified using commercial chemistries with fluorescently-labeled probes and unlabelled primers, depending on the real-time PCR instrument employed by investigators.

Using real-time PCR, cDNA is amplified further by fluorescent-labeled probes and unlabelled primers. Digital PCR is an alternative method that is useful for low viral loads, as well as during the initial spread or decline of a virus. The detection progress of four SARS-CoV-2 RT-qPCR primer-probes, including CDC-N1, CCDC-N, E-Sarbeco, and N-Sarbeco towards various water samples was systematically compared by Zhang et al. [35]. The CCDC-N primer-probe was found to be the most reliable primer-probe set for quantifying SARS-CoV-2 in monitoring by WBE, while CDC-N1 had the highest sensitivity for SARS-CoV-2 presence in wastewater with minimal COVID-19 occurrence.

The surveillance of wastewater with droplet digital PCR (ddPCR) is a comprehensive, cost-effective, and highly sensitive method to detect specific variants of the SARS-CoV-2 coronavirus in communities, allowing scientists and public health officials to track the spread and intervene of the virus [36]. The ddPCR takes wastewater testing to the next level, with the sensitivity and precision required to properly detect the presence of certain changes in wastewater samples, even at extremely low concentrations. Using ddPCR to detect WBE six days before clinical testing allows scientists to detect the virus with a sensitivity of one infected person in 10,000, which is crucial given that only 32% of those sick seek medical assistance. The utilization of ddPCR in identifying SARS-CoV-2 variants is illustrated in Figure 4 **(b)**. It can accurately detect and measure various SARS-CoV-2 variants within a single sample using a single-well test. Multiplexing in ddPCR generates fewer artifacts and provides easier data analysis compared to other techniques. As a result, ddPCR can effectively determine the copy number of both wild-type and variant genomes [36]. Researchers utilized droplet digital polymerase chain reaction (ddPCR) to evaluate viral enteric pathogens in untreated greywater and combined wastewater from three decentralized collection systems, which is the first quantitative report of its kind. Compared to standard qPCR, ddPCR allowed for sufficient sensitivity to quantify viruses in greywater. As illustrated in Figure 4**(c)**, Norovirus GII was detected in 96% of combined wastewater samples using either PCR method, with a density range of 5.2–7.9 log10 genome copies/L. Pathogen log reduction targets (LRTs) in non-potable water systems are determined based on an epidemiology-based model. The results support prior LRTs and consequent quantitative microbiological risk assessments (QMRA) of decentralized water reuse. [37].

### Using an electrochemical immunosensor

**Figure 5.**
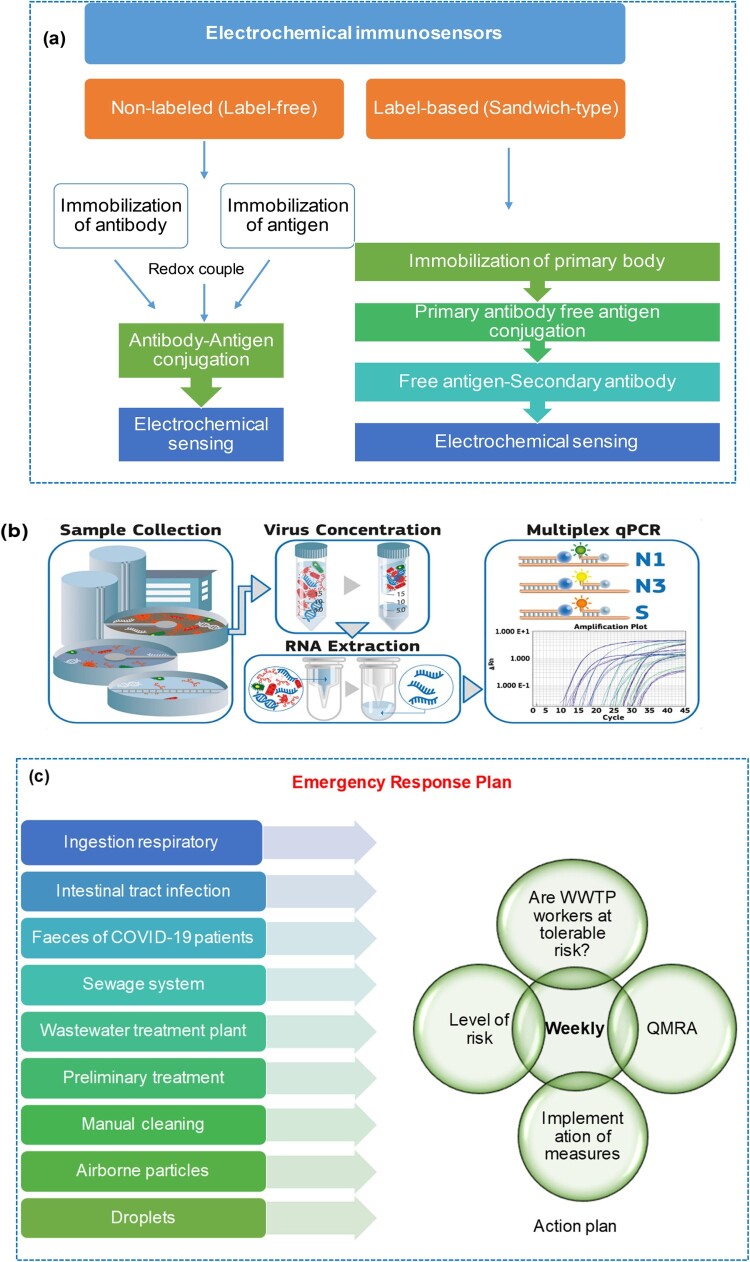
**(a)** Key formats for developing electrochemical immunosensors. **(b)** Detection of SARS-CoV-2 in wastewater using Multiplex quantitative PCR. **(c)** QMRA risk evaluation for workers in WWTPs.

The use of electrochemical immunosensors as an alternative method of detecting viruses in body fluids has been described by Lu et al. [38]. Figure 5 **(a)** demonstrates that biosensors contain receptors, detectors, and transducers, and require antibodies and antigens to react with the target analyte for detection. The biological variations are then transformed into a detectable physicochemical signal. Electrochemical immunosensors are particularly promising because they can detect pathogens in various mediums and multiple targets simultaneously. Unlike nucleic acid extraction, electrochemical immunoassays use ligand-based biological reactions to produce measurable electrochemical signals. An electrochemical immunoassay targets a virus species-specific antigen or an immune system antibody against it. Many studies have classified traditional electrochemical immunosensor construction into two categories: label-free and label-based (sandwich-type). For example, a new competitive electrochemical immunosensor for the detection of the Middle East Respiratory Syndrome Corona Virus (MERS-CoV) was developed by Lyqah et al. group [39] using an array of carbon electrodes modified with gold nanoparticles. It was successfully applied to spiked nasal samples. Due to their rapid detection, ease of downsizing, and potential on-site detection, electrochemical immunosensors may be more practical than PCR. Electrochemical immunosensors for detecting viral antigens have mostly been developed in nasal and saliva fluids. We know of three early studies that used immunological approaches to expose viruses in wastewater samples. Two common formats for electrochemical immunosensors are label-free and sandwich. The preparation of the label-free format is relatively easy. In terms of viral detection, it is comparable to and in some cases superior to the sandwich-type immunosensor. Regardless of the virus detection method, the pretreatment and concentration of wastewater samples are critical due to their complexity and dilute viral content. Pretreatment and concentration procedures for electrochemical immunoassay include electrostatic membrane adsorption, PEG precipitation, and ultracentrifugation. To improve future SARS-CoV-2 detection in wastewater, both sensitivity and practicability must be considered. Overall, the electrochemical immunosensor approach holds great promise for SARS-CoV-2 WBE. To quantify SARS-CoV-2 in wastewater samples, electrochemical immunosensors may be more practical than traditional PCR, given their rapid detection, miniaturization, and potential on-site measurement [38].

### Using a RT-LAMP

Loop-mediated isothermal amplification (LAMP) amplifies nucleic acid in isothermal environments, removing the need for thermal cycles. This approach uses six independent primers and is efficient, quick, and precise. Reverse transcription LAMP (RT-LAMP) techniques have been established for virus detection [40]. This has helped to detect early COVID-19 infections. RT-LAMP for WBE has been used sparingly so far. Optimization of RT-LAMP for COVID-19 WBE monitoring using SARSCoV-2 detection in wastewater could significantly reduce WBE costs. RT-LAMP techniques were used to successfully amplify SARS-CoV-2 in synthetic RNA, with a limit of detection (LOD) of up to 10 copies per 25 µL reaction (0.4 copies/L reaction volume). The speed with which the data are provided is one of the fundamental concepts essential for disease monitoring; excluding sample processing, the RT-LAMP method produces results in 35 min, as shown in **Table S2**. The LOD determined with synthetic RNA using the RT-LAMP method indicates a delicate method compared to a LOD of 118.6 copies/25 µl using digital PCR (dPCR). Huang el al.[41] achieved a more sensitive LOD of 2 copies/25 µl using RT-LAMP. However, the focus of this study was on the development of RT-LAMP for the clinical diagnosis of COVID-19. The fluorescent RT-LAMP procedure took less time to achieve positive amplification than the colorimetric RT-LAMP procedure. The two RT-LAMP techniques detected SARS-CoV-2 in wastewater samples differently. The colorimetric method had a prevalence of 12.5% compared to 31% for fluorescent RT-LAMP. When the initial template was increased, both colorimetric and fluorescent RT-LAMP techniques yielded similar results, with the latter having a higher prevalence. Increasing the concentration of the target nucleic acid on LOD reduces the time for positive amplification. RT-ddPCR can be used to determine the absolute concentration of SARS-CoV-2 in samples and verify the RT-LAMP results. RT-ddPCR had a higher prevalence (100%) than the two RT-LAMP techniques, but the concentration of SARS-CoV-2 per millilitre of wastewater was low. Based on RT-ddPCR results, both RT-LAMP procedures amplified SARS-CoV-2 in samples with less than 10 copies per 25 l. This suggests that RT-LAMP can detect smaller copies of viral RNA. The RTLAMP procedures proposed in this study could be used in many situations. These findings suggest that RT-LAMP could be used to detect SARS-CoV-2 in wastewater. So, with more research and optimization, RT-LAMP could replace WBE.

### Using a multiplex quantitative PCR

Multiplex PCR enables the detection and quantification of multiple gene targets in one sample, as depicted in Figure 5 **(b)**. This technique provides greater information with minimal sample volume, reducing pipetting-related variability and saving time and resources. However, due to its complexity, multiplex PCR assays are often fine-tuned to eliminate primer/probe set interactions or competition for shared reagents. Only a few papers have been published on multiplex RT–PCR for environmental testing. One of these discoveries occurred during the COVID-19 pandemic when microbial contamination of linen masks was discovered. Three target genes (N1, N3, and spike (S)) are routinely used for SARS-CoV-2 detection using multiplex PCR [21]. The N1 gene, followed by N2 and N3, is the most frequently used indicator for the detection of SARS-CoV-2 in wastewater samples [21, 42]. Because important mutations occur in this gene, the S gene was chosen to see if it could be used to distinguish between variants in the future. It was found to be useful. After testing the multiplex on a real wastewater sample by Navarro et al. [43], it was found that it has the same sensitivity as the single step. Using target genes and variations in gene copy number, the multiplex technique was used to determine and confirm changes in sensitivity over three months (December 2020 - February 2021). These findings demonstrated that the multiplex method is a robust, fast, reliable, and inexpensive method to monitor SARS-CoV-2 in wastewater samples [43].

### Rolling circle amplification (RCA) assays

TSou et al. [44] conducted a study demonstrating a nucleic acid detection technique that can identify SARS-CoV-2 in household sewage samples without the need for reverse transcription (Figure S3). The method uses multi-branch rolling circle amplification (mBRCA), an isothermal amplification technique that eliminates the need for reverse transcription and a thermal cycler. Rolling circle amplification (RCA) technology has already been used in the development of paper-based and electrochemical biosensors for the detection of SARS-CoV-2 RNA from clinical samples. Numerous biosensors have been newly developed to detect SARS-CoV-2 proteins in clinical samples. Alafeef et al. [45] have shown that their recently developed biosensor, in combination with a pattern recognition algorithm, offers several advantages. These advantages include high sensitivity (LOD of 1.52 copies/μL), selectivity in identifying SARS-CoV-2 over other species (such as H1N1 virus, SARS-CoV-1, E.coli, B.subtilis, and S.mutans) due to differences in fluorescence response patterns, and elimination of the need for an antibody.

### Identifying SARS-CoV-2 variants of concern (VOC)

Wastewater-based epidemiology has the potential to provide an additional method to monitor SARS-CoV-2 through surveillance. Nevertheless, accurately measuring and detecting the variants of concern (VOC) remains a major challenge with current techniques. Peccia et al. [46] conducted a study in the New Haven metropolitan area in Connecticut, USA, using quantitative reverse transcription-polymerase chain reaction (qRT-PCR) with N1 and N2 primer sets corresponding to those used for individual COVID-19 assays to measure the levels of SARS-CoV-2 RNA in primary sewage sludge. The results suggest that SARS-CoV-2 concentrations in primary sludge could serve as a useful metric for imposing or lifting infection control measures in areas where clinical testing capacity is limited or there are delays in reporting tests. The results demonstrate the value of monitoring viral RNA in municipal wastewater for population-level surveillance of SARS-CoV-2 infection. In communities where there is a delay between sampling and reporting of test results, timely wastewater data could provide significant warning of infection trends.

Wastewater-based genomic surveillance has the potential to improve community prevalence estimates and detect emerging viral variants, but there are two limiting factors to consider. These include sequence data of low quality and challenges in estimating the relative abundance of lineages in mixed samples. Karthikeyan et al. [2] have developed a tool called Freyja to estimate the relative abundance of virus lineages in mixed samples and capture the full range of virus diversity in community biospecimens. Freyja estimates the relative abundance of virus lineages in mixed samples and is designed to represent each SARS-CoV-2 lineage in the global phylogeny using a “barcode” library of lineage-defining mutations. The authors addressed key challenges by conducting a comprehensive 295-day sequencing project using both wastewater and clinical samples. The project was conducted in a controlled setting at a large university campus as well as in the surrounding county. They developed and used improved virus concentration techniques and deconvolution software that effectively differentiated between multiple strains of the virus found in wastewater. Identification of new strains of concern in wastewater samples occurred nearly two weeks earlier than clinical genomic surveillance and revealed several cases of virus transmission not detected by conventional methods. This research provides a practical approach to genomic surveillance of wastewater that facilitates the timely detection of SARS-CoV-2 variants and cryptic transmission.

It is strongly recommended that the genetic diversity of SARS-CoV-2 be tracked, as the diversifying selection may lead to the emergence of novel variants resistant to both natural and vaccine-induced immunity. In their studies. Smyth et al. [47] uncovered the presence of several cryptic lineages of SARS-CoV-2 in the New York City metropolitan area that were not detected by conventional clinical surveillance. Although they were unable to determine the origin of these lineages, their findings suggest that they have extended their receptor range, indicating the possibility of spread to an animal reservoir. Although other animal reservoirs were identified, no single animal was strongly represented in the rRNA sequencing analysis, suggesting that it is unlikely that a single animal reservoir is the source of all hidden lineages. In addition, they observed that these hidden lineages have acquired significant resistance to some neutralizing monoclonal antibodies derived from patients. They have also noticed that both WNY lineages and the Omicron VOC have a high number of mutated loci, which could be the result of convergent evolution to the shared selective pressure of antibody-mediated neutralization. Therefore, these cryptic lineages of SARS-CoV-2 may pose a public health problem and require further investigation. From December 2020 to February 2022, Amman et al. [48] analyzed 3,413 wastewater samples from 94 municipal catchments covering more than 59% of the Austrian population, using deep sequencing. The variant quantification system, VaQuERo, was designed to be robust and allowed to accurately determine the abundance of predefined variants in complex wastewater samples, providing spatiotemporal data. The data confirmed the accuracy of these findings by cross-referencing them with epidemiological records of over 311,000 individual cases. This study also revealed a higher level of viral genetic diversity during the Delta variant period and presented a method for predicting emerging variants. Additionally, they calculated variant-specific reproduction numbers from wastewater samples to determine the reproductive advantage of variants of concern. This study highlights the effectiveness of using wastewater-based epidemiology (WBE) on a national scale as a means to support public health, particularly in countries where individual surveillance is not widely available. In a study, D’Agostino et al. [24] proposed a PCR-based allelic- discrimination assay panel that can identify SARS-CoV-2 genotypes in various samples, including wastewater. The study confirmed that the assay can identify mutations in up to ten viral genome positions simultaneously. A pre-amplification phase was incorporated, in which a PCR reaction was performed with all genotype assays in a mixture, allowing the detection of variants at low levels (qPCR Ct values up to 38.5) in wastewater samples.

The detection of SARS-CoV-2 in wastewater in different countries is shown in Table 1.

**Table 1.** Global studies of SARS-CoV-2 prevalence in wastewater and waterways.

Places and regions of study	Water sample	Detection of viral RNA	Remarks	Reference
Queensland in Australia	Untreated wastewater	• (2/9) 22.2%		[49]
Noakhali in Bangladesh	Wastewater that includes toilets, passage drains, sewage waste tank	• 75.0% (12/16)		[50]
Minas Gerais, Rio de Janeiro, Santa Catarina, Rio Grande do Sul in Brazil	Treated effluents, Raw sewage, sewer network, hospital wastewater, rivers	• Wastewater: 12.5–• Rivers: 100.0% and 44.4%	Viral isolation with non-cultivable virus or detectable results. Epidemiological and clinical data support wastewater detection.	[51]
Gatineau, Ottawa in Canada	Primary clarified sludge (PCS), Influent post grit solids (PGS)	PCS samples: 92.7% and 90.6% PGS samples: 79.2 and 82.3%, With RT-qPCR to detect the N1 and N2 genes PGS samples: 92.7 and 90.6%.	RT-qPCR quantifies SARS-CoV-2 in PCS better than RT-ddPCR. Epidemiological data and Gene copies/L and had significant correlations after normalization.	[52]
Santiago in Chile	Effluent, Influent, and wastewater treatment plants.	• 100.0%.	No SARS-CoV-2 RNA was found in March-April 2020. The SARS-CoV-2 viral load from May to June, grew gradually	[53]
Wuhan in China	Influent, raw sewage, septic tanks, effluent wastewater	Sewage samples: 39.3% to 60% Effluents: 0%– 63.6%	SARS-CoV-2 was not found in disinfected effluents. The virus was isolated and found to be non-detectable.	[24]
Czech Republic	Raw wastewater	• 11.6%		[54]
Quito in Ecuador	Rivers	• 100.0%	Clinical and epidemiological data supported wastewater detection.	[55]
Montpellier, Paris in France	Effluent wastewater, treated wastewater, raw sewage, influent	Raw wastewater: 100.0% Treated wastewater: 75.0%	Clinical and epidemiological data supported wastewater detection. The viral load reached a peak after a 2-log increase, followed by a sharp decrease. The detection of RNA came before the exponential growth of the epidemic. After the lockdown, mid-June samples revealed 50-fold higher copies of the SARS-CoV-2 RNA genome, about a month after the lockdown was lifted. Within 2–3 weeks, early detection of SARS-CoV-2 RNA in wastewater signalled an increase in the number of new cases of COVID-19, which coincided with the exponential growth of epidemics.	[56]
North Westphalia in Germany	Wastewater, sludge	• 100.0% (Sanger sequencing)	The RNA gc/mL of SARS-CoV-2 was one log greater in the solid phase than in the liquid phase in influents. The total cases of COVID-19 reported in each area of the catchment was related to the total gene load in the wastewater. Isolation of viruses: negative	[57]
Jaipur in India	Effluent, influent and wastewater, hospital wastewater	• Influent: 75.0%–100.0%	In untreated wastewater, no RNA was found. In 10–14 days, primary recognistion of RNA in wastewater led an growth in reported cases.	[16]
Tel Aviv in Israel	Hospital effluents, raw sewage	• 38.4%		[29]
Monza, Rome, Milan, Bologna/Emilia Romagna, and Turin/Piedmont in Italy	Rivers, raw and treated wastewater	• Wastewater: 50.0%–100.0%	All samples taken from polluted rivers came back positive. Viral RNA in treated wastewater was undetectable. No detectable viruses were isolated during the viral isolation process. Early detection: RNA was found in wastewater before the first reported cases were discovered. Towards the end of 2019, SARS-CoV-2 was found to be in circulation in northern Italy. The first is on 18 December 2019 in Milan and Turin (retrospective study)	[58]
Toyama, Ishikawa, Yamanashi in Japan	secondary-treated wastewater, Influent, rivers	Secondary treated wastewater: 20.0% Influences: 20.0%–57.0%	The rivers did not have any SARS-CoV-2 RNA. The detection of wastewater was performed under low COVID-19 conditions.	[17]
Apeldoorn, Amsterdam, Hague, Schiphol airport, Tilburg Utrecht in Netherlands	Wastewater samples	• 68.9%.	The presence of RNA in wastewater predicted the appearance of the first clinical case between 4 and 6 days.	[21]
Islamabad in Pakistan	Sewage	• 27.0% (21/78)		[59]
Murcia, Lorca, Molina de Segura, Barcelona, Cartagena, Santiago de Compostela, Valencia, Cieza, Totana in Spain	Influents, raw sewage, sludge, secondary treated effluent, tertiary treated effluent, frozen archival samples from 2018 to 2020	Wastewater: 40.0–83.0% Sludge: 40.0% Secondary treated water: 11.0%	No positive tertiary effluent samples were found. Early detection of RNA in wastewater was found to be associated with clinical cases 12–16 days before they occurred.	[60]
Istanbul in Turkey	Wastewater, primary and activated sludge	• Primary and activated sludge: 100.0%.		[61]
Massachusetts, Connecticut, Virginia, Montana, Louisiana, New York in the USA	Raw sewage, daily primary sludge, influents, wastewater treatment plants	• 13.0%–100.0%	After exponentially increasing from March to April, viral titres in wastewater reached their highest point at the beginning of April and then began to decline. RNA detection in wastewater was consistent with clinical and epidemiological data. The early detection of RNA in wastewater was found to occur 1–10 days before the onset of COVID-19 cases.	[25]

To determine the risks to human health associated with exposure to pathogens, the quantitative microbial risk assessment (QMRA) method [62] (Figure 5 **(c))** has been used. QMRAs are important to evaluate respiratory exposure associated with wastewater, particularly relevant to the aerosolization of wastewater and the subsequent unintentional contamination of wastewater workers with SARS-CoV-2 during normal work tasks. The estimated median infection risk of SARS-CoV-2 QMRA in South China estimated median risk of infection after 1 h of exposure to the market was 2.23 × 10^−5^ [63]. A QMRA is applied for three COVID-19 scenarios, i.e. moderate, aggressive, and extreme, to study the effects of different stages of the pandemic. Figure 5 **(c)** demonstrates that QMRA investigations can pinpoint the distinct attributes of SARS-CoV-2, enabling the identification of public health and regulatory issues. Moreover, QMRA can address various queries regarding SARS-CoV-2, such as (i) whether a 2-meter social distancing measure provides adequate protection, (ii) the recommended air exchange/ventilation rate, and (iii) the necessary surface disinfection targets [28]. For SARS-CoV-2, QMRA revealed that aggressive and extreme scenarios resulted in an estimated risk of infection for workers that exceeded the tolerable risk. During COVID-19 and future pandemics, a QMRA of SARS-CoV-2 can be used as an early health warning tool.

## Inactivation of virus/ prevention

**Figure 6.**
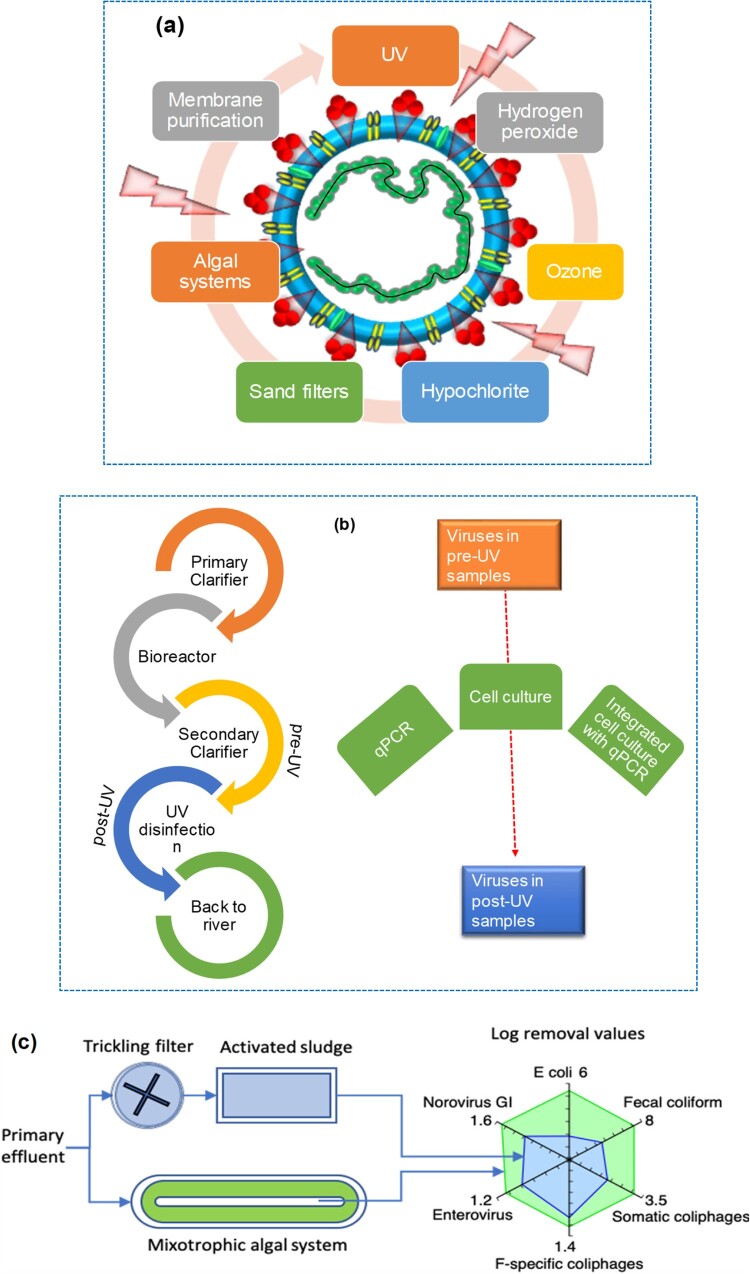
**(a)** Disinfection methods for efficient inactivation of the SARS-CoV-2 virus in wastewater. **(b)** The UV inactivation scheme of human infectious viruses in two large-scale wastewater treatment plants in Canada. **(c)** Schematic representation of an algal-based wastewater treatment system (the figure was adapted from Delanka-Pedige et al. [69] with permission).

Inactivation of SARS-CoV-2 is a crucial preventive measure against COVID-19. Most viruses are highly stable even when exposed to harsh environmental conditions. Higher temperatures, high or low pH, and sunlight help to kill viruses. The effectiveness of traditional disinfectants on the virus is influenced by several factors. SARS-CoV-2 was found to be unstable in the presence of disinfectants and at temperatures higher than 20°C, and thus can be effectively inactivated by various chemical disinfectants in wastewater treatment plants, including non-hazardous chlorine-containing hypochlorite or bleach, as well as chlorine oxide in combination with hydrogen peroxide, as shown in Figure 6 **(a)**. The effect of chlorine-containing disinfectants, quaternary ammonium compounds (QAC), ethanol, and heat on the inactivation of SARS-CoV-2 was studied by Xiling et al.[64]. This work showed that QAC disinfectants have high efficiency, short reaction times, and low dose effects. At very low concentrations, commercially available DNB was found to be highly effective in inactivating SARS-CoV-2. Ultraviolet (UV) irradiation, chlorination, and ozonation are widely used disinfection processes in water treatment.

### UV inactivation, chlorine treatment, and ozone treatment

Kong et al. [65]. reviewed the inactivation effects of UV irradiation, ozonation, and chlorination on SARS-CoV-2. Chlorine and UV light were effective against viruses, achieving a 2.5-3 log10 reduction (LR) in coliphage and E. coli. Sand-filtered samples exhibited better performance and demonstrated a reduction in different virus types, as shown in Figure 6 **(b)** for the UV inactivation scheme of human infectious viruses at two large-scale wastewater treatment plants in Canada [66].

Ozonation and chlorination were found to be effective in inactivating the viruses, as most coronaviruses are UV insensitive [65].

The US EPA has issued disinfection contact time (CT) values for ozone-medicated virus inactivation in the surface water treatment rule (SWTR) (US EPA 1989). The CT concept measures the efficiency of water disinfection. The disinfectant concentration (ozone) is represented by C, and the contact time is represented by T. Sigmon et al. [67] calculated CT values for several viruses in wastewater. In this study, the log reduction values (LRV) ranged from 1.0–1.32 for all viruses at pH 7.6 and 16 °C. These findings show that all organisms were 4-log inactivated at a CT of 1.0 mg min/L. Sewage treatment plant effluent has more viruses because enteric viruses are not adequately inactivated in wastewater effluent.

### Sand filters

In wastewater treatment, sand filters are frequently used for particle removal and have a virus removal capacity of less than 1 log_10_. Moringa Oleifera water extract was recently used by Samineni et al. [68] who developed functionalized sand filters Using the MS2 bacteriophage virus as a test, the produced sand reduced the virus concentration by 7 log_10_. MS2 specifically binds to certain seed components. It indicates that the functionalized sand gradually becomes saturated and needs to be regenerated or replaced in some way.

### Algal systems for virus removal

The use of algae-based wastewater treatment systems for virus removal is illustrated in Figure 6 **(c)**. Studies have shown that these systems are comparable to activated sludge in reducing the number of viruses [69]. This approach reduces the need for chlorination. Viral particles attach to algae and are subsequently removed through sedimentation, aided by higher temperatures, degradation of algal nucleic acids and proteins, and breakdown through exposure to sunlight.

### Membranes for wastewater virus removal

Based on membrane pore size and pore distributions, widely used membrane filtration methods are reverse osmosis (RO), nanofiltration (NF), ultrafiltration (UF), and microfiltration (MF) (**Figure S2**). Because the size of SARS-CoV-2 is approximately 100nm, membranes that remove it include reverse osmosis, nanofiltration, and ultrafiltration. The reported removal values using polymeric and ceramic membranes were very different as the values obtained ranged from 0.2–7.0 LRV. Although the EPA reports that reverse osmosis has suggestive log removals greater than 6, it is rarely used to remove pathogens from water. To remove particles and pollutants, RO can be combined with pretreatment. For MS2-coliphage, the membrane bioreactor and reverse osmosis systems had 2.3–2.9 log_10_ virus removal efficiency [70], which is lower than Vickers et al. [71]. MS2 is 60 times smaller than SARS-CoV-2, therefore, its removal efficiency should be better. The removal of noroviruses from raw sewage has been reported using a membrane bioreactor and a reverse osmosis system with sand-anthracite filters. With a log_10_ removal of 6 or 7, the system was efficient enough to keep the virus concentration below detection.

The pores of a nanofiltration membrane typically measure less than 10 nm in size, making it smaller than viruses. Although the literature on viral removal by nanofiltration is limited, numerous studies show log clearance rates ranging from 3 to 8 for various viruses. The efficacy of ultrafiltration (UF) in the removal of viruses is better documented than that of other membrane-based technologies. Its use for the removal of bacteria and viruses was recently reviewed [72]. The use of coagulant along with ultrafiltration improved the removal of the MS2 bacteriophage by 6.8–7.5 log_10_ by adjusting the pH of the secondary effluent. PP7 bacteriophage was removed using a 67-nm polyethersulfone ultrafiltration membrane. Log_10_ removal ranged from 1.5–2.8. As a result of electrostatic interactions, divalent cations in feed solutions are less efficient at viral elimination. A 150 kDa polyethersulfone membrane with grafted zwitterionic polymer hydrogels lowered water permeability while providing 3–4 Log_10_ removal of the human viruses MS2 and HAdV-2. Asymmetric ultrafiltration membranes have been evaluated for viral elimination using pore interconnectivity. Membranes with higher interconnectivity had stronger virus retention under a range of operating conditions. Because the pores of the microfiltration membrane are larger than 100 nm, they remove protozoa and bacteria better than viruses. It showed a 3 log_10_ MS2 reduction with only a 22% flux reduction.

O’ Brien et al. report that membrane bioreactors can achieve virus removal of 7 log_10_ under the right conditions [73]. The presence of slime on the membrane reduced the flux of water and prevented the penetration of the virus. The membrane is backwashed to maintain the water fluxes. The results of using harmless bacteriophages likely overestimate viral removal efficiencies. A membrane bioreactor plant can remove three viral families with an LR of 2.3–4.5 at pH 4. Virus removal was done using hydrophobic ceramic capillary membranes. Hexyl and octyl triethoxysilanes were used to increase the hydrophobicity of ceramic membranes. The log_10_ removal efficiency improved from ∼3.4 for the pristine membrane to 9.0 for the functionalized membrane. Viruses adsorb to membrane material as a result of hydrophobic interactions. In this case, the membranes would become saturated with viruses and need to be cleaned. In-line coagulation of polyaluminum chloride with ceramic membrane filtration achieved 6.7 log_10_ virus removal for bacteriophage Qβ [74]. Many nanomaterials have been used to remove or deactivate viruses. The viruses were removed from the water using a nanocomposite filter system containing silver nanotubes. A 7 LR in MS2 was achieved using Cu_2_O-coated multiwall nanotube filters at pH 5. A Cu_2_O-coated multiwall nanotube membrane removed 4 log_10_ MS2 at pH 5–9 [75].

To remove viruses, ultrafiltration, reverse osmosis, and nanofiltration membranes must be perfect. Membranes can be mechanically damaged during the filtration process. Several reviews of the separation membrane's integrity and the structure's property relationships are available. Due to the lack of sensitive detection methods, regulatory agencies only credit high-pressure membranes with 2 log_10_ virus removal, while researchers show that it reaches 7 log_10_. A system that monitors membranes’ integrity in pulses has been proposed for real-time virus removal. This fluorescent-based method is 4 log_10_ sensitive. A diagnosis decision tree could help determine the types of imperfections in a membrane. Nanoparticle counting had the lowest LRV values. Aqueous polyvinyl alcohol was used to fill nanoscale membrane flaws selectively. The plugs were stabilized with citric acid, sodium hydroxide, and EDTA [76].

## Limitations

Any epidemiological indicator has biases and limitations. More research with more comprehensive epidemiological data is needed before using wastewater as an early-warning system to better assess its potential. Epidemiological data were limited in the early days of the pandemic due to several factors, including limitations in testing capacity and changes in testing guidance over time. Sampling for WBE applications faces challenges such as sampling time, viral decay, dilution, and contamination from disinfectants and detergents that enter the sewage system. Optimal recovery and concentration methods for enveloped viruses must be investigated. The development of improved extraction and purification methods, in conjunction with next-generation sequencing and metaviromics, will be required to revitalize viral wastewater screening. Changing demographics present a challenge; therefore, normalization of the population is essential for WBE. Wastewater monitoring requires lockdown and small population sampling. Stigmatizing and fear-based behaviours of infected individuals may lead to distrust in health services. The virus can be spread by concealing cases, home treatment, and increased risk of infecting family members and then the community. All published studies used various technical approaches for wastewater sampling, sample concentration, and RNA detection. It is also critical to compare the results of various analytical techniques.

Sequencing viruses from wastewater samples is technically challenging due to the low concentration of target molecules, the degradation of nucleic acids, and the numerous interfering factors in the wastewater matrix. This is in contrast to sequencing viruses from epidemiological case samples. The emergence of new SARS-CoV-2 variants with higher transmissibility and/or immune evasion properties presents a future challenge for controlling the spread of the virus. Factors such as antiviral immunity from natural infection or vaccines, social behaviour, viral reservoirs, testing strategies, and global transmission make it difficult to predict how the virus will evolve in the future. These dynamics of SARS-CoV-2 evolution pose a major challenge to containment efforts. The current pandemic has led to a significant increase in interest in WBE. However, several technical, scientific, and policy challenges must be overcome to fully realize its potential. To accurately quantify and interpret data, a better understanding of the amount and duration of viral shedding via feces is needed, especially considering the impact of patient immune status and variant-specific characteristics. The ability to translate WBE findings into actionable public health measures promptly will be critical [48].

It should be noted that the targeted sequencing approach used in some studies may not identify mutations outside the specific region targeted. While whole genome sequencing of wastewater has been used in some cases, the results have often been inconclusive. Since wastewater contains virions shed from multiple infected individuals, mutations identified through sequencing cannot be definitively attributed to a specific genome. Additionally, it has not been possible to isolate viable viruses from wastewater, making it impossible to sequence single virus genotypes. Another challenge in sequencing SARS-CoV-2 genomes from wastewater is uneven coverage. This can result in inadequate sequencing of phylogenetically and clinically significant regions of the genome with adequate coverage levels [47].

## Conclusions and recommendations

Effluent monitoring is a comprehensive health assessment tool. Thousands of wastewater treatment plants around the world can successfully monitor the health of billions of people. Key biological markers of bacteria, viruses, and fungi can indicate disease in a population. However, before wastewater virus monitoring can be used as an effective early warning technique for the entire population, several challenges must be overcome. When quality assurance/quality controls are in place, sewage virus surveillance can be used in conjunction with clinical diagnostics. Real-time surveillance of wastewater viruses as an early warning system requires regular (daily) sampling, rapid sample delivery, analysis, and reporting. For molecular target detection, high efficiency and sensitivity are required due to the complexity of the sample matrix and low concentrations of virus components. Several recent studies have used real-time qPCR to identify the N, E, and S genes of SARS-CoV-2 RNA isolated and concentrated from wastewater and sewage sludge. Constituents in complex wastewater and sewage sludge samples can inhibit RT-qPCR reactions, resulting in low sensitivity and/or false negative results. Extraction of viruses and viral components from wastewater and sewage sludge samples is difficult. The inhibition of RT-qPCR by wastewater and sewage sludge sample matrix is crucial, as it varies significantly depending on the location of sample collection. Many published studies on the surveillance of SARS-CoV-2 wastewater virus have lacked recovery assays to assess how well SARS-CoV-2 and/or its RNA can be collected, condensed and extracted. The recovery and extraction efficiency of surrogate viruses from the same wastewater samples varied. Apart from the concentration of surrogate viruses, the recovery and extraction efficiency may also be affected by the chemical composition of the wastewater. Therefore, despite its weaknesses, a surrogate virus should be used to assess the validity of wastewater virus monitoring. Researchers can compare the results of different experiments using a uniform surrogate and a consistent virus dose. During a monitoring period, the number and characteristics of individuals contributing to the wastewater flow may change. Normalization of effluent flow and the study population is usually required if virus concentrations are to be compared over time. Therefore, many published studies do not normalize using human feces, making it difficult to determine the prevalence of infection in the population using viral RNA concentrations in wastewater samples. Without correcting for wastewater flow and the actual number of people contributing to wastewater, the concentrations of SARS-CoV-2 RNA in wastewater cannot be effectively compared over time and between communities. To ensure that WBE can be used reliably and meaningfully, SARS-CoV-2 must be detected using methods and procedures for collecting, concentrating, extracting, and standardizing wastewater samples. Most modern SARS-CoV-2 tests use a “quantitative polymerase chain reaction with reverse transcription” (RT -qPCR). This approach can only detect known RNA viruses. To continue looking for new viruses in the future, two conditions must be met. First, the viruses must be excreted into wastewater via feces, urine, saliva, blood, and sputum from Covid-19 patients. Second, their genome is needed so that RT -qPCR reagents can be tailored to them. By using high-throughput sequencing and bioinformatics, unknown virus genomes can be identified.

## Supplementary Material

Supplemental Material

## References

[1] Corpuz MVA, Buonerba A, Zarra T, et al. Advances in virus detection methods for wastewater-based epidemiological applications. Case Stud Chem Environ Eng. 2022;6:100238, doi:10.1016/j.cscee.2022.100238 37520925 PMC9339091

[2] Karthikeyan S, Levy JI, De Hoff P, et al. Wastewater sequencing reveals early cryptic SARS-CoV-2 variant transmission. Nature. 2022;609(7925):101–108. doi:10.1038/s41586-022-05049-6 35798029 PMC9433318

[3] Uddin M, Mustafa F, Rizvi TA, et al. SARS-CoV-2/COVID-19: viral genomics, epidemiology, vaccines, and therapeutic interventions. Viruses. 2020;12(5):526, doi:10.3390/v12050526 32397688 PMC7290442

[4] Tang A, Tong Z-D, Wang H-L, et al. Detection of novel coronavirus by RT-PCR in stool specimen from asymptomatic child, China. Emerging Infect Dis. 2020;26(6):1337, doi:10.3201/eid2606.200301 PMC7258461 32150527

[5] Li X, Kulandaivelu J, Guo Y, et al. SARS-CoV-2 shedding sources in wastewater and implications for wastewater-based epidemiology. J Hazard Mater. 2022;432:128667, doi:10.1016/j.jhazmat.2022.128667 35339834 PMC8908579

[6] Bogler A, Packman A, Furman A, et al. Rethinking wastewater risks and monitoring in light of the COVID-19 pandemic. Nat Sustainability. 2020;3(12):981–990. doi:10.1038/s41893-020-00605-2

[7] Gundy PM, Gerba CP, Pepper IL. Survival of coronaviruses in water and wastewater. Food Environ Virol. 2008;1(1):10.

[8] Wong SCC, Chan JKC, Lee KC, et al. Development of a quantitative assay for SARS coronavirus and correlation of GAPDH mRNA with SARS coronavirus in clinical specimens. J Clin Pathol. 2005;58(3):276, doi:10.1136/jcp.2004.016592 15735160 PMC1770583

[9] Wang X-W, Li J-S, Guo T-K, et al. Concentration and detection of SARS coronavirus in sewage from Xiao Tang Shan Hospital and the 309th Hospital. J Virol Methods. 2005;128(1-2):156–161. doi:10.1016/j.jviromet.2005.03.022 15964082 PMC7112879

[10] Wu F, Zhang J, Xiao A, et al. Data-Driven models reveal mutant cell behaviors important for myxobacterial aggregation. mSystems. 2020;5(4):e00614–20. doi:10.1128/mSystems.00518-20 32665330 PMC7363006

[11] Majumder A, Gupta AK, Ghosal PS, et al. A review on hospital wastewater treatment: A special emphasis on occurrence and removal of pharmaceutically active compounds, resistant microorganisms, and SARS-CoV-2. J Environ Chem Eng. 2021;9(2):104812, doi:10.1016/j.jece.2020.104812 33251108 PMC7680650

[12] Wang X-W, Li J-S, Jin M, et al. Study on the resistance of severe acute respiratory syndrome-associated coronavirus. J Virol Methods. 2005;126(1):171–177. 15847934 10.1016/j.jviromet.2005.02.005 PMC7112909

[13] Wang J, Feng H, Zhang S, et al. SARS-CoV-2 RNA detection of hospital isolation wards hygiene monitoring during the Coronavirus Disease 2019 outbreak in a Chinese hospital. Int J Infect Dis. 2020;94:103–106. doi:10.1016/j.ijid.2020.04.024 32311449 PMC7165090

[14] Zhang D, Ling H, Huang X, et al. Potential spreading risks and disinfection challenges of medical wastewater by the presence of Severe Acute Respiratory Syndrome Coronavirus 2 (SARS-CoV-2) viral RNA in septic tanks of Fangcang Hospital. Sci Total Environ. 2020;741:140445, doi:10.1016/j.scitotenv.2020.140445 32599407 PMC7308756

[15] Kam K-Q, Yung CF, Cui L, et al. A well infant with coronavirus disease 2019 with high viral load. Clin Infect Dis. 2020;71(15):847–9. 32112082 10.1093/cid/ciaa201 PMC7358675

[16] Kumar M, Patel AK, Shah AV, et al. First proof of the capability of wastewater surveillance for COVID-19 in India through detection of genetic material of SARS-CoV-2. Sci Total Environ. 2020;746:141326. 32768790 10.1016/j.scitotenv.2020.141326 PMC7386605

[17] Kitajima M, Iker BC, Rachmadi AT, et al. Quantification and genetic analysis of salivirus/klassevirus in wastewater in Arizona, USA. Food Environ Virol. 2014;6(3):213–216. doi:10.1007/s12560-014-9148-2 24863500

[18] Rimoldi SG, Stefani F, Gigantiello A, et al. Presence and infectivity of SARS-CoV-2 virus in wastewaters and rivers. Sci Total Environ. 2020;744:140911, doi:10.1016/j.scitotenv.2020.140911 32693284 PMC7358170

[19] Shi J, Li X, Zhang S, et al. Enhanced decay of coronaviruses in sewers with domestic wastewater. Sci Total Environ. 2022;813:151919, doi:10.1016/j.scitotenv.2021.151919 34826473 PMC8610560

[20] John DE, Rose JB. Review of factors affecting microbial survival in groundwater. Environ Sci Technol 2005;39(19):7345–7356. doi:10.1021/es047995w 16245801

[21] Medema G, Heijnen L, Elsinga G, et al. Presence of SARS-coronavirus-2 RNA in sewage and correlation with reported COVID-19 prevalence in the early stage of the epidemic in The Netherlands. Environ Sci Technol Lett. 2020;7(7):511–516. doi:10.1021/acs.estlett.0c00357 37566285

[22] Li X, Zhang S, Shi J, et al. Uncertainties in estimating SARS-CoV-2 prevalence by wastewater-based epidemiology. Chem Eng J. 2021;415:129039, doi:10.1016/j.cej.2021.129039 33642938 PMC7896122

[23] Sharkey ME, Kumar N, Mantero AMA, et al. Lessons learned from SARS-CoV-2 measurements in wastewater. Sci Total Environ 2021;798:149177, doi:10.1016/j.scitotenv.2021.149177 34375259 PMC8294117

[24] D’Agostino Y, Rocco T, Ferravante C, et al. Rapid and sensitive detection of SARS-CoV-2 variants in nasopharyngeal swabs and wastewaters. Diagn Microbiol Infect Dis 2022;102(4):115632, doi:10.1016/j.diagmicrobio.2021.115632 35074623 PMC8719921

[25] Hart OE, Halden RU. Computational analysis of SARS-CoV-2/COVID-19 surveillance by wastewater-based epidemiology locally and globally: Feasibility, economy, opportunities and challenges. Sci Total Environ. 2020;730:138875, doi:10.1016/j.scitotenv.2020.138875 32371231 PMC7175865

[26] Li X, Kulandaivelu J, Zhang S, et al. Data-driven estimation of COVID-19 community prevalence through wastewater-based epidemiology. Sci Total Environ. 2021;789:147947, doi:10.1016/j.scitotenv.2021.147947 34051491 PMC8141262

[27] Jahn K, Dreifuss D, Topolsky I, et al. Early detection and surveillance of SARS-CoV-2 genomic variants in wastewater using COJAC. Nat Microbiol. 2022;7(8):1151–1160. doi:10.1038/s41564-022-01185-x 35851854 PMC9352586

[28] Ahmed W, Bertsch PM, Bivins A, et al. Comparison of virus concentration methods for the RT-qPCR-based recovery of murine hepatitis virus, a surrogate for SARS-CoV-2 from untreated wastewater. Sci Total Environ. 2020;739:139960, doi:10.1016/j.scitotenv.2020.139960 32758945 PMC7273154

[29] Brouwer AF, Eisenberg JNS, Pomeroy CD, et al. Epidemiology of the silent polio outbreak in Rahat, Israel, based on modeling of environmental surveillance data. Proc Natl Acad Sci USA. 2018;115(45):E10625–E10E33. 30337479 10.1073/pnas.1808798115 PMC6233100

[30] Gerba CP, Betancourt WQ, Kitajima M. How much reduction of virus is needed for recycled water: A continuous changing need for assessment? Water Res 2017;108:25–31. doi:10.1016/j.watres.2016.11.020 27838026 PMC7112101

[31] Ahmed W, Kitajima M, Tandukar S, et al. Recycled water safety: Current status of traditional and emerging viral indicators. Curr Opin Environ Sci Health. 2020;16:62–72. doi:10.1016/j.coesh.2020.02.009

[32] La Rosa G, Bonadonna L, Lucentini L, et al. Coronavirus in water environments: Occurrence, persistence and concentration methods - A scoping review. Water Res 2020;179:115899, doi:10.1016/j.watres.2020.115899 32361598 PMC7187830

[33] Balboa S, Mauricio-Iglesias M, Rodriguez S, et al. The fate of SARS-COV-2 in WWTPS points out the sludge line as a suitable spot for detection of COVID-19. Sci Total Environ. 2021;772:145268, doi:10.1016/j.scitotenv.2021.145268 33556806 PMC7980226

[34] Corman VM, Landt O, Kaiser M, et al. Detection of 2019 novel coronavirus (2019-nCoV) by real-time RT-PCR. Eurosurveillance. 2020;25(3):2000045. 31992387 10.2807/1560-7917.ES.2020.25.3.2000045 PMC6988269

[35] Zhang S, Li X, Shi J, et al. Analytical performance comparison of four SARS-CoV-2 RT-qPCR primer-probe sets for wastewater samples. Sci Total Environ. 2022;806:150572, doi:10.1016/j.scitotenv.2021.150572 34582851 PMC8464025

[36] Heijnen L, Elsinga G, de Graaf M, et al. Droplet digital RT-PCR to detect SARS-CoV-2 signature mutations of variants of concern in wastewater. Sci Total Environ. 2021;799:149456, doi:10.1016/j.scitotenv.2021.149456 34371414 PMC8332926

[37] Jahne MA, Brinkman NE, Keely SP, et al. Droplet digital PCR quantification of norovirus and adenovirus in decentralized wastewater and graywater collections: Implications for onsite reuse. Water Res 2020;169:115213, doi:10.1016/j.watres.2019.115213 31671297 PMC7017454

[38] Lu D, Zhu DZ, Gan H, et al. Prospects and challenges of using electrochemical immunosensors as an alternative detection method for SARS-CoV-2 wastewater-based epidemiology. Sci Total Environ. 2021;777:146239, doi:10.1016/j.scitotenv.2021.146239

[39] Layqah LA, Eissa S. An electrochemical immunosensor for the corona virus associated with the Middle East respiratory syndrome using an array of gold nanoparticle-modified carbon electrodes. Mikrochim Acta. 2019;186(4):224, doi:10.1007/s00604-019-3345-5 30847572 PMC7088225

[40] Wong Y-P, Othman S, Lau Y-L, et al. Loop-mediated isothermal amplification (LAMP): a versatile technique for detection of micro-organisms. J Appl Microbiol 2018;124(3):626–643. doi:10.1111/jam.13647 29165905 PMC7167136

[41] Huang WE, Lim B, Hsu C-C, et al. RT-LAMP for rapid diagnosis of coronavirus SARS-CoV-2. Microb Biotechnol. 2020;13(4):950–961. doi:10.1111/1751-7915.13586 32333644 PMC7264870

[42] Hamouda M, Mustafa F, Maraqa M, et al. Wastewater surveillance for SARS-CoV-2: Lessons learnt from recent studies to define future applications. Sci Total Environ 2021;759:143493, doi:10.1016/j.scitotenv.2020.143493 33190883 PMC7648500

[43] Navarro A, Gómez L, Sanseverino I, et al. SARS-CoV-2 detection in wastewater using multiplex quantitative PCR. Sci Total Environ 2021;797:148890, doi:10.1016/j.scitotenv.2021.148890 34298359 PMC8278834

[44] Tsou J-H, Liu H, Stass SA, et al. Rapid and sensitive detection of SARS-CoV-2 using clustered regularly interspaced short palindromic repeats. Biomedicines. 2021;9(3):239. 33673601 10.3390/biomedicines9030239 PMC7997215

[45] Alafeef M, Dighe K, Moitra P, et al. Monitoring the viral transmission of SARS-CoV-2 in still waterbodies using a lanthanide-doped carbon nanoparticle-based sensor array. ACS Sustain Chem Eng. 2022;10(1):245–258. doi:10.1021/acssuschemeng.1c06066 35036178

[46] Peccia J, Zulli A, Brackney DE, et al. Measurement of SARS-CoV-2 RNA in wastewater tracks community infection dynamics. Nat Biotechnol 2020;38(10):1164–1167. doi:10.1038/s41587-020-0684-z 32948856 PMC8325066

[47] Smyth DS, Trujillo M, Gregory DA, et al. Tracking cryptic SARS-CoV-2 lineages detected in NYC wastewater. Nat Commun. 2022;13(1):635, doi:10.1038/s41467-022-28246-3 35115523 PMC8813986

[48] Amman F, Markt R, Endler L, et al. Viral variant-resolved wastewater surveillance of SARS-CoV-2 at national scale. Nat Biotechnol 2022;40(12):1814–1822. doi:10.1038/s41587-022-01387-y 35851376

[49] Ahmed W, Angel N, Edson J, et al. First confirmed detection of SARS-CoV-2 in untreated wastewater in Australia: A proof of concept for the wastewater surveillance of COVID-19 in the community. Sci Total Environ. 2020;728:138764, doi:10.1016/j.scitotenv.2020.138764 32387778 PMC7165106

[50] Ahmed F, Islam MA, Kumar M, et al. First detection of SARS-CoV-2 genetic material in the vicinity of COVID-19 isolation centre through wastewater surveillance in Bangladesh. medRxiv. 2020;8(18):20194696.

[51] Aguiar-Oliveira MDL, Campos A, R. Matos A, et al. Wastewater-Based epidemiology (WBE) and viral detection in polluted surface water: A valuable tool for COVID-19 surveillance—A brief review. Int J Environ Res Public Health. 2020;17(24):9251, doi:10.3390/ijerph17249251 33321987 PMC7764684

[52] D’Aoust PM, Mercier E, Montpetit D, et al. Quantitative analysis of SARS-CoV-2 RNA from wastewater solids in communities with low COVID-19 incidence and prevalence. Water Res. 2021;188:116560, doi:10.1016/j.watres.2020.116560 33137526 PMC7583624

[53] Ampuero M, Valenzuela S, Valiente-Echeverria F, et al. SARS-CoV-2 detection in sewage in Santiago, Chile-preliminary results. MedRxiv. 2020;7:2020-07.

[54] Mlejnkova H, Sovova K, Vasickova P, et al. Preliminary study of SARS-CoV-2 occurrence in wastewater in the Czech Republic. Int J Environ Res Public Health. 2020;17(15):5508, doi:10.3390/ijerph17155508 32751749 PMC7432771

[55] Guerrero-Latorre L, Ballesteros I, Villacrés-Granda I, et al. SARS-CoV-2 in river water: Implications in low sanitation countries. Sci Total Environ. 2020;743:140832, doi:10.1016/j.scitotenv.2020.140832 32679506 PMC7343659

[56] Wurtzer S, Marechal V, Mouchel J-M, et al. Evaluation of lockdown impact on SARS-CoV-2 dynamics through viral genome quantification in Paris wastewaters. MedRxiv. 2020. doi:10.1101/2020.04.12.20062679 PMC7812418 33334397

[57] Westhaus S, Weber F-A, Schiwy S, et al. Detection of SARS-CoV-2 in raw and treated wastewater in Germany – Suitability for COVID-19 surveillance and potential transmission risks. Sci Total Environ. 2021;751:141750, doi:10.1016/j.scitotenv.2020.141750 32861187 PMC7434407

[58] La Rosa G, Iaconelli M, Mancini P, et al. First detection of SARS-CoV-2 in untreated wastewaters in Italy. Sci Total Environ. 2020;736:139652, doi:10.1016/j.scitotenv.2020.139652 32464333 PMC7245320

[59] Sharif S, Ikram A, Khurshid A, et al. Detection of SARs-CoV-2 in wastewater, using the existing environmental surveillance network: an epidemiological gateway to an early warning for COVID-19 in communities. MedRxiv. 2020. doi:10.1101/2020.06.03.20121426

[60] Randazzo W, Cuevas-Ferrando E, Sanjuán R, et al. Metropolitan wastewater analysis for COVID-19 epidemiological surveillance. Int J Hyg Environ Health. 2020;230:113621, doi:10.1016/j.ijheh.2020.113621 32911123 PMC7462597

[61] Kocamemi BA, Kurt H, Sait A, et al. SARS-CoV-2 detection in Istanbul wastewater treatment plant sludges. MedRxiv. 2020. doi:10.1101/2020.05.12.20099358

[62] Zaneti RN, Girardi V, Spilki FR, et al. Quantitative microbial risk assessment of SARS-CoV-2 for workers in wastewater treatment plants. Sci Total Environ. 2021;754:142163, doi:10.1016/j.scitotenv.2020.142163 32911141 PMC7468340

[63] Zhang X, Ji Z, Yue Y, et al. Infection risk assessment of COVID-19 through aerosol transmission: a case study of south China seafood market. Environ Sci Technol 2021;55(7):4123–4133. doi:10.1021/acs.est.0c02895 32543176

[64] Xiling G, Yin C, Ling W, et al. In vitro inactivation of SARS-CoV-2 by commonly used disinfection products and methods. Sci Rep. 2021;11(1):2418, doi:10.1038/s41598-021-82148-w 33510320 PMC7843590

[65] Kong J, Lu Y, Ren Y, et al. The virus removal in UV irradiation, ozonation and chlorination. Water Cycle. 2021;2:23–31. doi:10.1016/j.watcyc.2021.05.001

[66] Qiu Y, Li Q, Lee BE, et al. UV inactivation of human infectious viruses at two full-scale wastewater treatment plants in Canada. Water Res 2018;147:73–81. doi:10.1016/j.watres.2018.09.057 30300783

[67] Sigmon C, Shin G-A, Mieog J, et al. Establishing surrogate–virus relationships for ozone disinfection of wastewater. Environ Eng Sci 2015;32(6):451–460. doi:10.1089/ees.2014.0496

[68] Samineni L, Xiong B, Chowdhury R, et al. 7 log virus removal in a simple functionalized sand filter. Environ Sci Technol. 2019;53(21):12706–12714. doi:10.1021/acs.est.9b03734 31593449

[69] Delanka-Pedige HMK, Munasinghe-Arachchige SP, Zhang Y, et al. Bacteria and virus reduction in secondary treatment: Potential for minimizing post disinfectant demand. Water Res 2020;177:115802, doi:10.1016/j.watres.2020.115802 32311576

[70] Prado T, Silva DM, Guilayn WC, et al. Quantification and molecular characterization of enteric viruses detected in effluents from two hospital wastewater treatment plants. Water Res 2011;45(3):1287–1297. doi:10.1016/j.watres.2010.10.012 21040941

[71] Vickers JC, Dummer M, Le T, et al. Removal of MS-2 coliphage in full-scale reverse osmosis systems. AWWA Water Science. 2019 2019;1(6):e1158.

[72] Al Aani S, Mustafa TN, Hilal N. Ultrafiltration membranes for wastewater and water process engineering: A comprehensive statistical review over the past decade. J Water Process Eng. 2020;35:101241, doi:10.1016/j.jwpe.2020.101241

[73] O’Brien E, Xagoraraki I. Removal of viruses in membrane bioreactors. J Environ Eng. 2020;146(7):03120007, doi:10.1061/(ASCE)EE.1943-7870.0001743

[74] Wattanachira L, Rakruam P, Yanthongyu P, et al. Bacteriophage removal efficiency of In-line coagulation with ceramic membrane filtration. Eng J. 2017;21(4):1–9. doi:10.4186/ej.2017.21.4.1

[75] Németh Z, Szekeres GP, Schabikowski M, et al. Enhanced virus filtration in hybrid membranes with MWCNT nanocomposite. R Soc Open Sci. 2019;6(1):181294. doi:10.1098/rsos.181294 30800376 PMC6366182

[76] Suzuki T, Okamura M, Niinae M. Plugging nanoscale imperfections in the polyamide active layer of thin-film composite reverse osmosis membrane to inhibit advective solute transport. Desalination. 2020;487:114506, doi:10.1016/j.desal.2020.114506

