# Supplementary material for: SARS-CoV-2 detection and inactivation in water and wastewater: review on analytical methods, limitations and future research recommendations
Source: Emerg Microbes Infect. 2023 Jun 21;12(2):2222850. doi: 10.1080/22221751.2023.2222850 (PMC10286680; doi:10.1080/22221751.2023.2222850)
Supplement: Supplemental Material [file TEMI_A_2222850_SM7323.docx]

**SARS-CoV-2 detection and inactivation in water and wastewater: Review on analytical methods, limitations and future research recommendations**

Parashuram Kallem*^1,2^, Hanaa Hegab^1,2^, Habiba Alsafar^3,4,5^, Shadi W. Hasan ^1,2^, Fawzi Banat*^1,2^

^1^Center for Membranes and Advanced Water Technology (CMAT), Khalifa University of Science and Technology, PO Box 127788, Abu Dhabi, United Arab Emirates

^2^Department of Chemical Engineering, Khalifa University of Science and Technology, PO Box 127788, Abu Dhabi, United Arab Emirates

^3^Center for Biotechnology (BTC), Khalifa University of Science and Technology, PO Box 127788, Abu Dhabi, United Arab Emirates

^4^Department of Biomedical Engineering, College of Engineering, Khalifa University of Science and Technology, Abu Dhabi, United Arab Emirates

^5^Emirates Bio-research center, Ministry of interior, Abu Dhabi, United Arab Emirates

*Corresponding authors:* [*fawzi.banat@ku.ac.ae*](mailto:fawzi.banat@ku.ac.ae)*;* [*parashuram.kallem@ku.ac.ae*](mailto:parashuram.kallem@ku.ac.ae)

| (a) 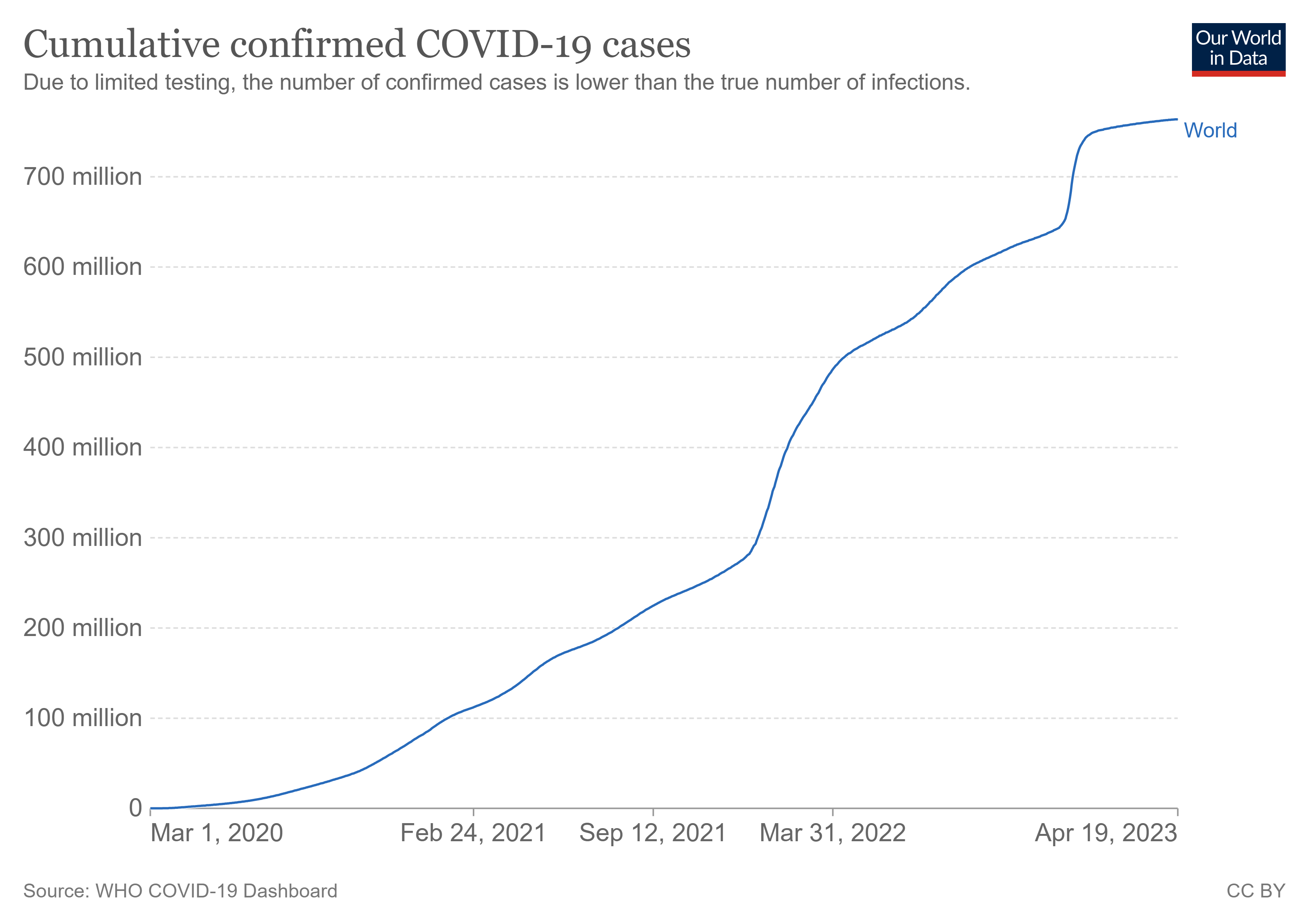 |
| --- |
| (b)  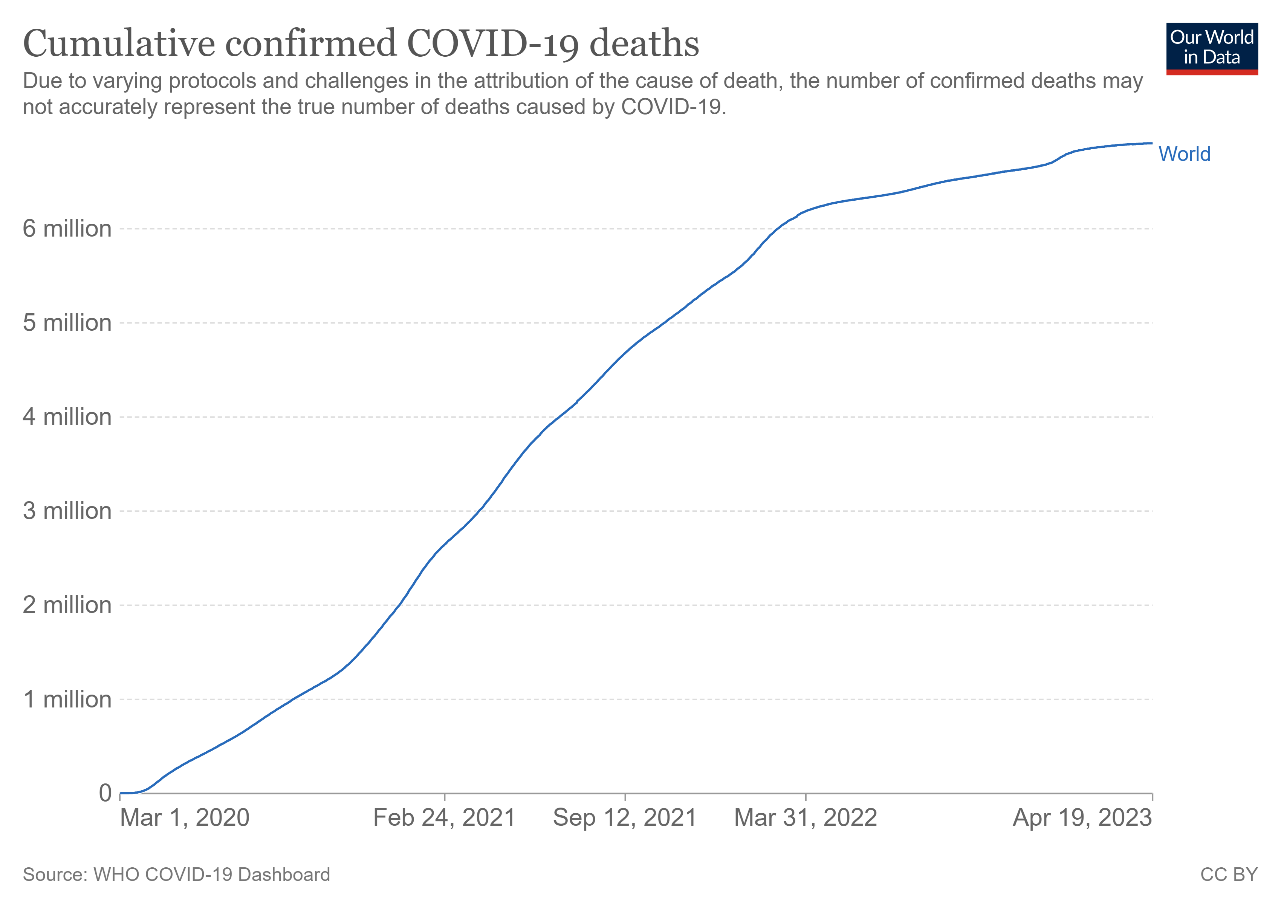 |

**Figure S1. (a-b)** As of 19 April 2023, global confirmed cases and deaths (data are from WHO COVID-19 Dashboard. Geneva: World Health Organization, 2023, Available online: <https://covid19.who.int/>).

**Table S1.** Various concentration methods reported from different locations of the world.

| **Concentration method** | **Type of water** | **Maximum concentration (copies/L)** | **Positive rate** | **Location** | **Reference** |
| --- | --- | --- | --- | --- | --- |
| PEG precipitation of centrifuged supernatant | Untreated wastewater  Treated wastewater | 0.05–1.87 × 10^5^ | 0/4  77%  7/9 | Wuchang Fangcang Hospital, China | [1] |
| Aluminum flocculation - Precipitation of beef extract | Untreated wastewater  Treated wastewater | N1: 1.4 × 10^5^  N2: 3.4 × 10^5^  N3: 3.1 × 10^5^  <2.5 × 10^5^ | N1: 21/42  N2: 23/42  N3: 27/42  Secondary: 2/18  Tertiary: 0/12 | Murcia, Spain | [2] |
| PEG precipitation of centrifugated supernatant | Untreated wastewater  Treated wastewater | 5.6 × 10  3.5 × 10^2^ | 8 May: 100%  27 May:100%  8 May: 0%  27 May: 0% | Ahmedabad, India | [3] |
| PEG precipitation of filtered samples | Untreated wastewater | N1: 10^4^–2 × 10^5^  N2: 3 × 10^4^–10^5^  N3: 10^4^–10^5^ | N1: 4/6  N2: 1/6  N3: 4/6 | Massachusetts, USA | [4] |
| Ultracentrifugation | Untreated wastewater  Treated wastewater | >10^7.5^  ~10^6^ | 23/23 (100%)  6/8 (75%) | Paris, France | [5] |
| PEG/dextran precipitation of centrifuged supernatant | Untreated wastewater | NA (PCR detection) | 100%  12/12 | Milan and Rome, Italy | [6] |
| Electronegative membrane-direct RNA extraction; ultrafiltration | Untreated wastewater | 1.2 × 10^3^ | 2/9 (22% | Brisbane, Australia | [7] |

**Table S2.** Limits of detection for RT-LAMP using the colorimetric method (Table adapted from Navarro et al. [8]).

| **RNA copies/reaction** | 10,000 | 1000 | 100 | 10 | 1 | NTC |
| --- | --- | --- | --- | --- | --- | --- |
| Number of positive reactions | 3/3 | 3/3 | 2/3 | 2/3 | 1/3 | 0 |
| Time for positive reaction (min) | 25 | 25 | 30 | 35 | 35 | N/A |


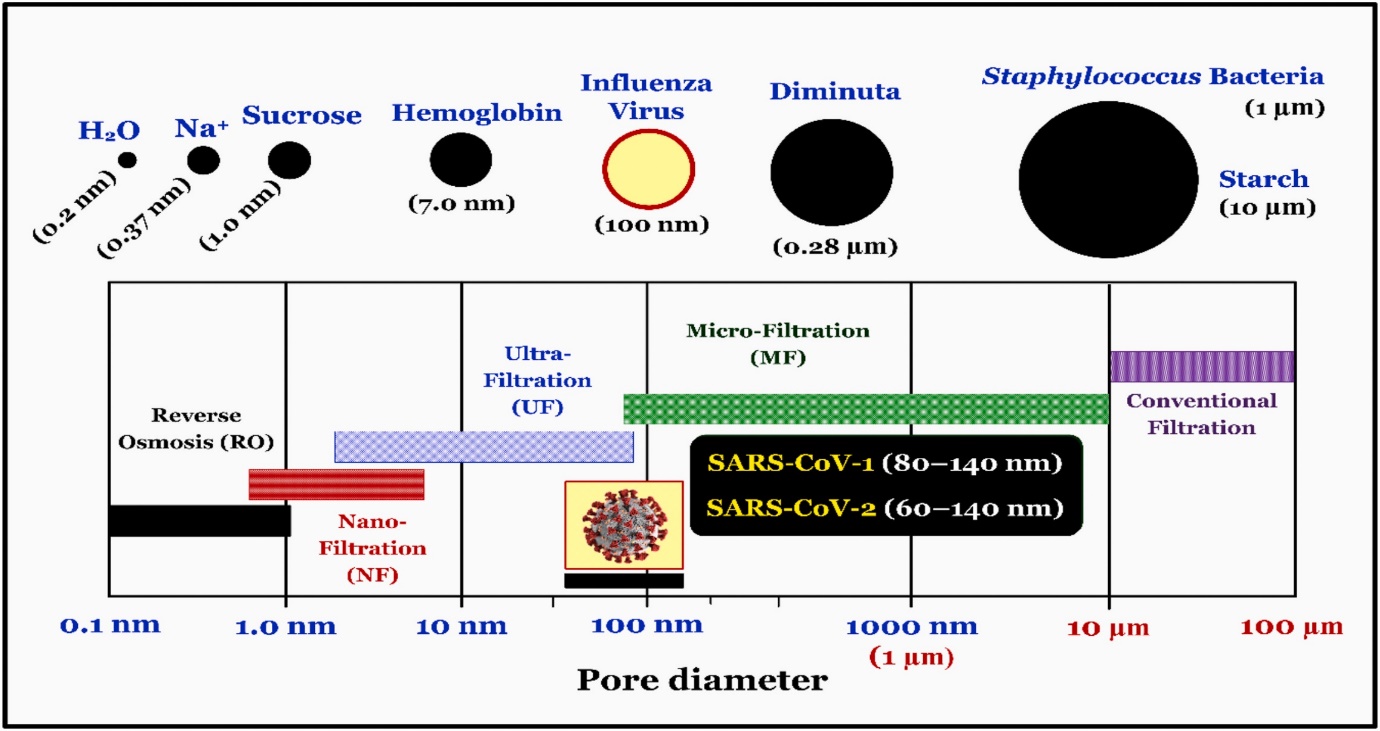


**Figure S2.**  Comparison of the sizes of microorganisms, including SARS-CoV-1 and SARS-CoV-2 coronaviruses, with the diameters of membrane pores [9].


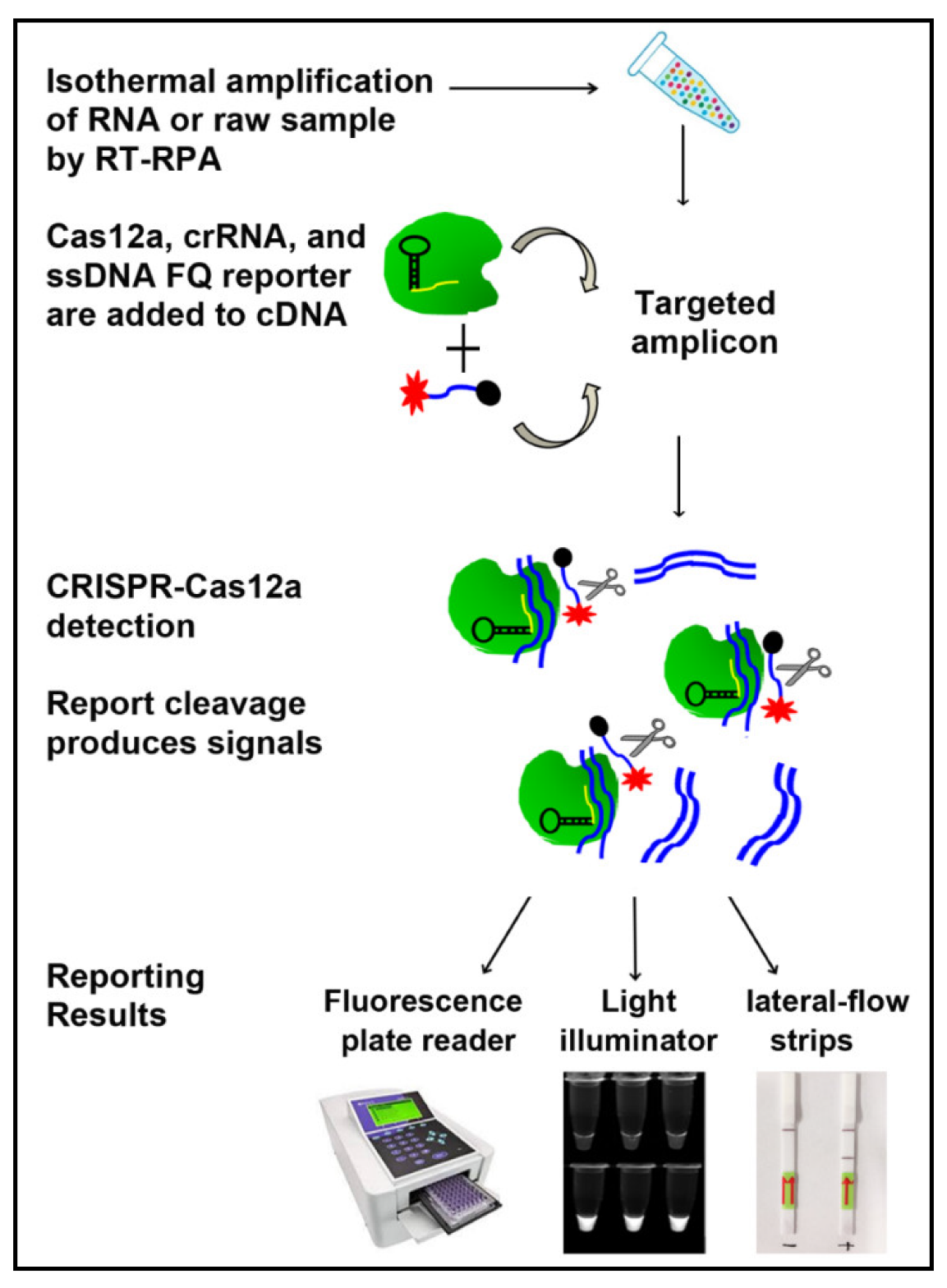


**Figure S3.** Overview of the clustered regularly interspaced short palindromic repeats (CRISPR)-Cas12a assay to detect SARS-CoV.

1. Zhang W, Du R-H, Li B, et al. Molecular and serological investigation of 2019-nCoV infected patients: implication of multiple shedding routes. *Emerging microbes & infections* 2020; 9: 386-389.

2. Randazzo W, Truchado P, Cuevas-Ferrando E, Simón P, Allende A, Sánchez G. SARS-CoV-2 RNA in wastewater anticipated COVID-19 occurrence in a low prevalence area. *Water Research* 2020; 181: 115942. DOI: 10.1016/j.watres.2020.115942

3. Kumar M, Patel AK, Shah AV, et al.: First proof of the capability of wastewater surveillance for COVID-19 in India through detection of genetic material of SARS-CoV-2. 2020; 2020.2006.2016.20133215.

4. Wu F, Zhang J, Xiao A, et al. SARS-CoV-2 titers in wastewater are higher than expected from clinically confirmed cases. *Msystems* 2020; 5: e00614-00620.

5. Wurtzer S, Marechal V, Mouchel J-M, Moulin L. Time course quantitative detection of SARS-CoV-2 in Parisian wastewaters correlates with COVID-19 confirmed cases. *MedRxiv* 2020.

6. La Rosa G, Iaconelli M, Mancini P, et al. First detection of SARS-CoV-2 in untreated wastewaters in Italy. *Science of The Total Environment* 2020; 736: 139652.

7. Ahmed W, Angel N, Edson J, et al. First confirmed detection of SARS-CoV-2 in untreated wastewater in Australia: A proof of concept for the wastewater surveillance of COVID-19 in the community. *Science of The Total Environment* 2020; 728: 138764. DOI: 10.1016/j.scitotenv.2020.138764

8. Navarro A, Gómez L, Sanseverino I, et al. SARS-CoV-2 detection in wastewater using multiplex quantitative PCR. *The Science of the Total Environment* 2021; 797: 148890. DOI: 10.1016/j.scitotenv.2021.148890

9. Tran HN, Le GT, Nguyen DT, et al. SARS-CoV-2 coronavirus in water and wastewater: A critical review about presence and concern. *Environmental Research* 2021; 193: 110265. DOI: 10.1016/j.envres.2020.110265
